# Supplementary material for: Public preferences towards data management and governance in Swiss biobanks: results from a nationwide survey
Source: BMJ Open. 2022 Aug 26;12(8):e060844. doi: 10.1136/bmjopen-2022-060844 (PMC9422864; doi:10.1136/bmjopen-2022-060844)
Supplement: Author's manuscript [file bmjopen-2022-060844.draft_revisions.pdf]

BMJ Open

BMJ Open is committed to open peer review. As part of this commitment we make the peer review history of every article we publish publicly available.

When an article is published we post the peer reviewers' comments and the authors' responses online. We also post the versions of the paper that were used during peer review. These are the versions that the peer review comments apply to.

The versions of the paper that follow are the versions that were submitted during the peer review process. They are not the versions of record or the final published versions. They should not be cited or distributed as the published version of this manuscript.

BMJ Open is an open access journal and the full, final, typeset and author-corrected version of record of the manuscript is available on our site with no access controls, subscription charges or pay-per-view fees (http://bmjopen.bmj.com).

If you have any questions on BMJ Open's open peer review process please email info.bmjopen@bmj.com

BMJ Open

Public preferences towards data management and governance in Swiss biobanks – results from a nationwide survey

Journal:	*BMJ Open*
Manuscript ID	bmjopen-2022-060844
Article Type:	Original research
Date Submitted by the Author:	19-Jan-2022
Complete List of Authors:	Brall, Caroline; ETH Zurich, Department of Health Sciences and Technology<br>Berlin, Claudia; University of Bern, Institute of Social and Preventive Medicine<br>Zwahlen , Marcel; University of Bern, Insitute of Social and Preventive Medicine<br>Vayena, Effy; ETH Zurich, Department of Health Sciences and Technology<br>Egger, Matthias; University of Bern, Institute of Social and Preventive Medicine, Bristol Medical School; University of Bristol, Population Health Sciences<br>Ormond, Kelly E.; ETH Zurich, Department of Health Sciences and Technology
Keywords:	PUBLIC HEALTH, Health policy < HEALTH SERVICES ADMINISTRATION & MANAGEMENT, Information management < BIOTECHNOLOGY & BIOINFORMATICS

SCHOLARONE™ Manuscripts

I, the Submitting Author has the right to grant and does grant on behalf of all authors of the Work (as defined in the below author licence), an exclusive licence and/or a non-exclusive licence for contributions from authors who are: i) UK Crown employees; ii) where BMJ has agreed a CC-BY licence shall apply, and/or iii) in accordance with the terms applicable for US Federal Government officers or employees acting as part of their official duties; on a worldwide, perpetual, irrevocable, royalty-free basis to BMJ Publishing Group Ltd ("BMJ") its licensees and where the relevant Journal is co-owned by BMJ to the co-owners of the Journal, to publish the Work in this journal and any other BMJ products and to exploit all rights, as set out in our licence.

The Submitting Author accepts and understands that any supply made under these terms is made by BMJ to the Submitting Author unless you are acting as an employee on behalf of your employer or a postgraduate student of an affiliated institution which is paying any applicable article publishing charge ("APC") for Open Access articles. Where the Submitting Author wishes to make the Work available on an Open Access basis (and intends to pay the relevant APC), the terms of reuse of such Open Access shall be governed by a Creative Commons licence – details of these licences and which Creative Commons licence will apply to this Work are set out in our licence referred to above.

Other than as permitted in any relevant BMJ Author's Self Archiving Policies, I confirm this Work has not been accepted for publication elsewhere, is not being considered for publication elsewhere and does not duplicate material already published. I confirm all authors consent to publication of this Work and authorise the granting of this licence.

For peer review only

Public preferences towards data management and governance in Swiss biobanks – results from a nationwide survey

Caroline Brall[1], Claudia Berlin[2], Marcel Zwahlen[2], Effy Vayena[1], Matthias Egger[2,3] and Kelly E. Ormond[1]

[1] Health Ethics and Policy Lab, Department of Health Sciences and Technology, ETH Zurich, Zurich, Switzerland
[2] Institute of Social and Preventive Medicine, University of Bern, Bern, Switzerland
[3] Population Health Sciences, Bristol Medical School, University of Bristol, Bristol, UK

Corresponding author: Caroline Brall, Hottingerstrasse 10, 8092 Zurich, caroline.brall@hest.ethz.ch

Word count: 4350

For peer review only

Abstract

Objectives: This article aims to measure the Swiss public's willingness to participate in personalised health research and their preferences regarding data management and governance.

Setting: Results from a nationwide survey of members of the Swiss public are presented.

Participants: 15,106 randomly selected Swiss residents received the survey in September 2019.The response rate was 34.1% (n = 5,156). Respondents' age ranged from 18 to 79 years, with fairly even spread across sex and age categories between 25 – 64 years.

Primary and secondary outcome measures: Willingness to participate in personalised health research and opinion regarding data management and governance

Results: Most respondents prefer to be contacted and re-consented for every new project using their data (39%, 95% CI: 37.4% - 40.7%), or state that their preference depends on the type of project being considered (29.4%, 95% CI: 27.9% - 31%). Additionally, a majority (52%, 95% CI: 50.3% - 53.8%) prefer their data or samples to be stored anonymously or in a coded form (43.4%, 95% CI: 41.7% - 45.1%). Of those who preferred their data to be anonymised, most also indicated that they want to be re-contacted for every new project (36.8%, 95% CI: 34.5% - 39.2%); these preferences are in conflict. Most respondents desire to personally own their data. Finally, most Swiss respondents trust their doctors and researchers at universities to protect their data.

Conclusion: These insights into the public's preferences allow Swiss biobanks and research institutions to organise their management and governance strategies according to the expectations and preferences of potential participants, which may vary by country. Models where participants can choose how they would like to interact with the process, while more complex, may increase individuals' willingness to provide their data to biobanks.

Strengths and limitations of this study

- This nationwide study examined preferences of 5,156 members of the Swiss public regarding data management and governance when hypothetically providing data or samples for personalised health research.
- We could construct non-response weights by all sociodemographic characteristics, making the results reasonably generalisable to the Swiss population.
- Respondents were asked about the type of consent and storage preferences in two separate questions, making it impossible to determine which one is more important to respondents.

For peer review only - http://bmjopen.bmj.com/site/about/guidelines.xhtml

Keywords: Public preferences, biobanks, data management, data governance, personalised health research, Switzerland

For peer review only

For peer review only - http://bmjopen.bmj.com/site/about/guidelines.xhtml

INTRODUCTION

Personalised health strives to improve clinical outcomes by providing more effective prevention and therapy for individuals, thus improving morbidity and mortality. Large amounts of health data and samples are needed to achieve the goals of personalised medicine, which requires both that individuals participate in biomedical research by donating their health data and samples, and that the samples and data may be reused in future research studies [1]. Studies have shown that public preferences for sharing personal health and genetic information in research settings vary depending on the societal, demographic and jurisdictional context in which the study takes place [2]. In Switzerland, we previously found that the public generally holds a positive opinion towards personalised health research and that a majority are hypothetically willing to donate data and samples for such research projects [3]. Yet, in biobank research, concerns about data privacy and reuse are closely linked to public willingness to participate in research [3–6].

Two research design components greatly influence the ability to reuse samples and data for future research and participants' willingness to provide samples [7]. First, a biobank can choose to store data in an identifiable, coded or anonymous form. If data and samples are stored and labelled with identifiable information, they can be easily linked back to donors. In contrast, anonymous data storage removes all personal identifiers from the data; linking back to a specific person is possible, but only with enormous effort. A middle option is storage in a coded form, which links persons' data and samples via a securely stored code. Second, how informed consent occurs can influence participant trust and the feasibility of sample and data reuse [8,9]. Broad consent (also referred to as general consent) denotes that individuals consent to their data and samples being used for research in future, non-specified studies. Broad consent approaches increase data availability, thereby enabling more future research, but rely on potential participants' comfort with agreeing to future research without knowing what areas it will address [10]. On the other hand, specific consent requires that individuals provide consent for every study that uses explicitly their data or samples. Besides the presented consent models various other more specific consent models exist, among them blanket consent, presumed consent with opt-out, or tiered consent (for a detailed description of such models see [11–13]). While study-specific consent is often technically challenging and resource-intensive to implement and update, it allows participants a sense of control over their samples and data, particularly when stigmatising or controversial research is conducted. Finally, design choices over data storage and consent approaches influence how data can be used in research and if individuals must and can be re-contacted for consent, and also influence the potential for reporting individual research results [14].

For peer review only - http://bmjopen.bmj.com/site/about/guidelines.xhtml

In Switzerland, the existing biobanks are mostly linked to the five university hospitals and generally use a broad consent approach [15,16]. Personalised health initiatives are scaling up in Switzerland, and broad consent has been promoted on the national level [16]. However, we do not know how the Swiss public views this type of consent and what other views and expectations they hold towards management and governance of their health data and samples. A better understanding of what potential participants expect from personalised health biobanks and related research infrastructures, and which data management and governance practices influence their willingness to donate data and samples, will allow biobanks to address these public needs at the early stages of project development.

This paper presents findings from a nationwide survey [3] about the Swiss public's preferences regarding data management and governance when hypothetically providing data or samples for personalised health research. We report preferences regarding types of consent, data storage, ownership and management responsibilities, and what information respondents perceive they would need before donating data or samples. Last, we present respondents' levels of trust in different actors of the health data ecosystem.

METHODS

We conducted a cross-sectional survey, with full details previously published regarding the survey development and pilot testing [3]. Briefly, the survey contained questions to assess attitudes, concerns and expectations towards (hypothetically) providing health data or biological samples for personalised health research. It consisted of six parts: general attitudes; motivations and barriers to participating in personalised health research; expectations towards data management; data governance; data sharing and uses; and willingness to receive results. The questionnaire was available in English, German, Italian and French and consisted of 23 closed (binary, 5-point Likert scale and multiple-choice) questions. This paper will focus on the findings for data management, data governance, and data sharing.

The survey was mailed to 15,106 Swiss residents over the age of 18 in September 2019. According to the Swiss Statistical Survey Ordinance, the Swiss Federal Statistical Office (FSO) provided the stratified random sample, which covered the three main language regions (SR 431.012.1, article 13c). The sampled potential participants received the survey by regular mail post in their language of correspondence (indicated at the municipality). They could complete the survey either by paper or through a web link on the Qualtrics survey platform (Qualtrics, Provo, Utah 84604, USA); both options allowed mapping of individual responses with sociodemographic characteristics provided by the FSO. Participants received two reminders

For peer review only - http://bmjopen.bmj.com/site/about/guidelines.xhtml

(the first after three weeks, the second after seven weeks). Responses were collected over 20 weeks between September 2019 and January 2020. We complied with data protection regulations at both research institutions (ETH Zurich and the University of Bern) and obtained ethics approval from the Ethics Committee of ETH Zurich (EK 2018-N-66).

Public involvement

The development of the research question and outcome measures was informed by finding out more about the public's preferences and expectations towards donating health data or samples for personalised health research. Members of the public were involved in testing preliminary versions of the questionnaire. The questionnaire was adapted according to their comments.
As we indicated to respondents that they will not be contacted again after filling out the survey, results will not be actively disseminated to the participants but will be available to respondents upon request. The results will furthermore be published open access.

Statistical analysis

After evaluating the data for completeness (minimum of 50% data completeness for inclusion in analysis), we analysed it in STATA (version 15, College Station, TX, USA). We linked the survey data with the demographic data from the FSO, including gender, age, language, household size, nationality, marital status, and the municipality of residence using the FSO unique identifiers. To account for differences between survey respondents and the general population of Switzerland, we applied survey weights using gender, age, language region. We included further variables provided by the FSO. Our final dataset included age, gender, nationality, number of household members, marital status, having biological children, language region, type of municipality of residence (urban or rural), education, religiosity, current or previous employment in the health sector, health status, and the type of survey response (online or paper-based). We used relative proportions for statistical analysis, as they allow a more straightforward interpretation than odds ratios: comparing a proportion of 60% to one of 30% leads to a relative proportion of 2.0, but an odds ratio of 3.5. We used modified multivariable Poisson regression, to analyse the relative proportion of respondents' willingness to provide health data or biological samples for personalised health research and to adjust for the other characteristics of respondents [17]. To simplify the interpretation of the analysis results, we collapsed a 5 point Likert scale (1 = no trust to 5 = strong trust) to binary variables (1-3 = low trust, 4-5 = strong trust).

For peer review only - http://bmjopen.bmj.com/site/about/guidelines.xhtml

RESULTS

Sample description

As reported in detail elsewhere [3], 5,086 complete responses were received, representing an overall response rate of 34.1%. Most responses (71.0%) were submitted via the web-based survey platform. Respondents' age ranged from 18 to 79 years, with fairly even spread across sex and age categories between 25 – 64 years (Table 1). The majority of respondents were Swiss nationals (76.0%), lived in a German-speaking region (70.8%), in urban areas (61.0%), were married (50.9%), had children (57.6%) and lived in 3-5 person households (43.7%). Most had secondary education (65.2%), described their health status as somewhat (47.1%) or very healthy (36.6%), and the majority had never worked in the health sector (79.8%). Thirteen per cent reported they were very religious. More than half of respondents (53.6%) expressed their hypothetical willingness to provide their health data or biological samples for personalised health research purposes [3].

Preferences on types of consent

Regarding consent to use data from a hypothetical biobank, 39.0% (95% CI: 37.4% - 40.7%) of respondents wanted to be asked for consent for each new project (study-specific consent). Further, 29.4% (95% CI: 27.9% - 31.0%) indicated that this would depend on the type of project, and 17.7% (95% CI: 16.4% - 19.0%) preferred to be asked only once when donating the data or samples (general consent). A minority of respondents (13.8%, 95% CI: 12.7% - 15.1%) were unsure of their preference. Compared to those who only wanted to be asked just once, participants who wanted to be asked for permission for each project or those who were unsure about this were less willing to hypothetically provide their data and samples for research (adjusted Relative Proportions (aRP) = 0.86, 95% CI: 0.79 - 0.93; aRP = 0.35, 95% CI: 0.29 - 0.43) (Figure 1). Table 1 shows demographic information for types of consent.

For peer review only - http://bmjopen.bmj.com/site/about/guidelines.xhtml

Table 1. Preferences on type of consent according to sociodemographic factors (all weighted proportions except N).

	Sample	Population	How often should biobank asked for permission?			
			Ask me only once, when I donate my data and/or samples	Ask me again for every new project	It would depend on the type of project that is being considered	Not sure
	N	%	% [95% CI]	% [95% CI]	% [95% CI]	% [95% CI]
**Total**	**5,086**	**100**	**17.7 [16.4,19.0]**	**39.0 [37.4,40.7]**	**29.4 [27.9,31.0]**	**13.8 [12.7,15.1]**
**Age group**						
18 - 24	594	8.6	10.8 [8.2,14.1]	41.8 [37.0,46.7]	34.7 [30.1,39.6]	12.7 [9.8,16.4]
25 - 34	758	17.7	14.5 [11.7,17.9]	41.8 [37.6,46.2]	30.2 [26.3,34.4]	13.4 [10.7,16.7]
35 - 44	632	18.5	15.4 [12.3,19.1]	46.8 [42.2,51.5]	26.3 [22.4,30.6]	11.5 [8.9,14.8]
45 - 54	927	19.5	18.2 [15.4,21.4]	41.3 [37.6,45.1]	27.5 [24.2,31.1]	13.0 [10.6,15.9]
55 - 64	1,005	17.5	17.4 [14.8,20.4]	37.6 [34.0,41.4]	29.6 [26.3,33.2]	15.3 [12.8,18.3]
65 - 74	857	13.1	26.8 [23.3,30.5]	27.4 [24.0,31.1]	29.7 [26.2,33.4]	16.1 [13.5,19.2]
75 - 79	313	5.2	25.1 [19.7,31.5]	21.3 [16.3,27.2]	35.5 [29.3,42.2]	18.1 [13.5,23.8]
Total	5,086	100	17.7 [16.4,19.0]	39.0 [37.4,40.7]	29.4 [27.9,31.0]	13.8 [12.7,15.1]
**Sex**						
Male	2,451	50.1	18.8 [17.0,20.8]	38.3 [35.9,40.8]	29.5 [27.3,31.8]	13.4 [11.8,15.2]
Female	2,635	49.9	16.6 [14.9,18.4]	39.8 [37.5,42.1]	29.4 [27.3,31.6]	14.3 [12.7,16.0]
Total	5,086	100	17.7 [16.4,19.0]	39.0 [37.4,40.7]	29.4 [27.9,31.0]	13.8 [12.7,15.1]
**Nationality**						
Swiss	4,216	76.0	18.6 [17.2,20.0]	39.3 [37.5,41.1]	29.9 [28.2,31.6]	12.3 [11.2,13.5]
Non-Swiss	870	24.0	15.0 [12.3,18.1]	38.2 [34.3,42.3]	28.1 [24.5,32.0]	18.7 [15.7,22.0]
Total	5,086	100	17.7 [16.4,19.0]	39.0 [37.4,40.7]	29.4 [27.9,31.0]	13.8 [12.7,15.1]
**No. of household members**						
1	760	18.1	18.1 [14.9,21.9]	36.4 [32.2,40.9]	31.4 [27.4,35.8]	14.0 [11.3,17.2]
2	1,854	35.5	19.5 [17.4,21.7]	37.8 [35.1,40.6]	29.9 [27.4,32.5]	12.7 [11.0,14.7]
3-5	2,347	43.7	16.2 [14.4,18.1]	41.2 [38.7,43.7]	28.2 [26.0,30.5]	14.4 [12.7,16.3]
6 persons and more	125	2.8	15.9 [9.3,25.7]	36.5 [27.2,47.0]	29.5 [20.7,40.1]	18.1 [11.1,28.1]
Total	5,086	100	17.7 [16.4,19.0]	39.0 [37.4,40.7]	29.4 [27.9,31.0]	13.8 [12.7,15.1]
**Marital status**						
single	1,669	35.3	13.0 [11.1,15.2]	44.8 [41.8,47.9]	30.0 [27.3,32.9]	12.2 [10.3,14.3]
married	2,733	50.9	19.8 [18.0,21.7]	37.2 [35.0,39.5]	28.1 [26.1,30.2]	14.8 [13.3,16.6]
widowed	150	3.1	22.7 [15.3,32.4]	22.2 [15.3,31.0]	34.0 [25.6,43.6]	21.0 [14.6,29.4]
divorced	534	10.7	21.9 [17.9,26.6]	33.1 [28.4,38.1]	32.3 [27.8,37.2]	12.7 [9.7,16.4]
Total	5,086	100	17.7 [16.4,19.0]	39.0 [37.4,40.7]	29.4 [27.9,31.0]	13.8 [12.7,15.1]
**Biological children**						
Yes	2,968	57.6	19.6 [17.9,21.4]	36.9 [34.8,39.1]	28.8 [26.8,30.8]	14.7 [13.3,16.4]
No	2,074	42.4	15.1 [13.3,17.1]	41.8 [39.1,44.5]	30.5 [28.0,33.1]	12.6 [11.0,14.5]
Missing	44					
Total	5,086	100	17.7 [16.4,19.0]	39.0 [37.3,40.7]	29.5 [28.0,31.1]	13.8 [12.7,15.1]
**Language region**						
German	2,257	70.8	17.9 [16.3,19.6]	40.0 [37.9,42.2]	29.9 [28.0,32.0]	12.2 [10.8,13.7]
French	1,366	24.5	17.3 [15.3,19.5]	35.7 [33.0,38.4]	28.3 [25.7,30.9]	18.8 [16.6,21.1]
Italian	1,463	4.7	16.9 [15.0,19.1]	41.7 [39.0,44.3]	27.9 [25.6,30.4]	13.5 [11.7,15.5]
Total	5,086	100	17.7 [16.4,19.0]	39.0 [37.4,40.7]	29.4 [27.9,31.0]	13.8 [12.7,15.1]
**Urban/rural municipality**						
urban	3,104	61.0	17.5 [15.9,19.3]	39.5 [37.3,41.7]	29.5 [27.5,31.6]	13.4 [12.0,15.0]

For peer review only

intermediary	1,086	21.6	17.5 [15.0,20.3]	38.8 [35.3,42.4]	28.7 [25.6,32.0]	15.0 [12.6,17.8]
rural	896	17.3	18.6 [15.7,21.9]	37.6 [33.8,41.6]	30.0 [26.4,33.8]	13.8 [11.3,16.8]
Total	5,086	100	17.7 [16.4,19.0]	39.0 [37.4,40.7]	29.4 [27.9,31.0]	13.8 [12.7,15.1]
**Education**						
Compulsory education or less	385	8.4	12.1 [8.7,16.6]	28.0 [22.8,33.8]	24.5 [19.6,30.2]	35.4 [29.7,41.5]
Upper secondary education	3,394	65.2	17.4 [15.9,19.0]	37.6 [35.5,39.6]	30.8 [28.9,32.8]	14.3 [12.9,15.8]
Tertiary education	1,283	26.4	20.1 [17.5,23.0]	46.2 [42.8,49.7]	27.8 [24.8,30.9]	5.9 [4.6,7.6]
Missing	24					
Total	5,086	100	17.7 [16.4,19.0]	39.1 [37.4,40.8]	29.5 [27.9,31.1]	13.8 [12.7,15.0]
**Religion**						
Very much	695	13.0	16.5 [13.3,20.2]	38.1 [33.6,42.8]	27.2 [23.3,31.6]	18.2 [14.9,22.0]
Somewhat	2,217	43.6	18.2 [16.3,20.2]	35.4 [33.0,38.0]	31.2 [28.8,33.6]	15.2 [13.4,17.2]
Not at all	2,138	43.5	17.7 [15.7,19.8]	42.8 [40.2,45.4]	28.5 [26.2,31.0]	11.0 [9.5,12.7]
Missing	36					
Total	5,086	100	17.7 [16.5,19.1]	39.0 [37.3,40.7]	29.5 [28.0,31.1]	13.8 [12.6,15.0]
**Working in health sector?**						
Yes	1,010	20.2	17.7 [15.0,20.8]	44.7 [40.9,48.6]	28.9 [25.5,32.5]	8.6 [6.7,11.0]
No	4,063	79.8	17.7 [16.3,19.2]	37.6 [35.8,39.5]	29.6 [27.9,31.4]	15.0 [13.7,16.4]
Missing	13					
Total	5,086	100	17.7 [16.5,19.1]	39.1 [37.4,40.8]	29.5 [27.9,31.1]	13.7 [12.6,14.9]
**Health status**						
Very unhealthy	61	0.8	20.9 [10.2,37.9]	28.1 [15.5,45.4]	26.0 [12.5,46.3]	25.1 [12.5,44.0]
Somewhat unhealthy	115	2.0	13.5 [7.9,22.2]	29.8 [20.6,41.1]	26.4 [17.5,37.7]	30.3 [21.2,41.2]
Neutral	661	13.5	17.5 [14.4,21.2]	33.0 [28.7,37.6]	31.0 [26.8,35.5]	18.5 [15.1,22.5]
Somewhat healthy	2,578	47.1	18.7 [16.8,20.7]	36.6 [34.3,39.0]	31.1 [28.9,33.4]	13.6 [12.1,15.4]
Very healthy	1,643	36.6	16.7 [14.6,18.9]	45.1 [42.2,48.0]	27.1 [24.6,29.8]	11.1 [9.4,13.0]
Missing	28					
Total	5,086	100	17.7 [16.4,19.0]	39.0 [37.4,40.7]	29.5 [27.9,31.1]	13.8 [12.6,15.0]
**Type of response**						
Web-based	3,519	71.0	17.6 [16.1,19.2]	41.7 [39.7,43.7]	29.0 [27.2,30.9]	11.7 [10.4,13.1]
Paper-based	1,567	29.0	17.9 [15.6,20.5]	32.0 [29.1,35.1]	30.6 [27.7,33.6]	19.5 [17.1,22.0]
Total	5,086	100	17.7 [16.4,19.0]	39.0 [37.4,40.7]	29.4 [27.9,31.0]	13.8 [12.7,15.1]

Figure 1 around here

Preferences on data storage

When asked how they prefer data to be stored, most respondents preferred their data or samples to be anonymised (52.0%, 95% CI: 50.3% - 53.8%) or stored and used in a coded form (43.4%, 95% CI: 41.7% - 45.1%). Only a few respondents (4.5%, 95% CI: 3.9% - 5.3%) preferred their data to be stored and used in an identifiable form (Figure 2). Respondents who preferred coded or identifiable data and sample storage were more willing to hypothetically donate data or samples (aRP: 1.57, 95% CI: 1.47 – 1.68; aRP: 1.67, 95% CI: 1.47 – 1.89) than those who preferred anonymised samples. Many respondents who would like their data to be stored

For peer review only - http://bmjopen.bmj.com/site/about/guidelines.xhtml

anonymously indicated that they preferred to be re-contacted for every new project (36.8%, 95% CI: 34.5% - 39.2%), or re-contacted depending on the new project type (26.5%, 95% CI: 24.4% - 28.6%).

Figure 2 around here

Information participants need before deciding to donate data and samples to a biobank

We asked all respondents to endorse the top three information pieces they would need to decide whether to donate data and samples to a biobank (Figure 3). While nearly all options had at least 25.0% of the participants endorse them, the most supported pieces of information were "the exact types of research which will be conducted" (57.1%) and "who has access to my data and samples" (48.3%). Only very few indicated that none of this information would help them to decide (7.4%). Respondents were more willing to hypothetically provide data or samples when they endorsed any of the following information: "the exact types of research which will be conducted", "the potential benefits and risks of donating my data and samples", "the way data and samples are stored, such as made anonymous, encoded or stored together with my name", and "the security measures to keep data and samples private and protected". The answer options "who will benefit from the research" and "none of this information would help me decide" were negatively associated with willingness to donate data or samples to a biobank.

Figure 3 around here

Importance of financial or other material compensation for data donation

Most respondents indicated that money or any other material compensation would not/ rather not be important (56.9%) for deciding whether to participate in a Swiss publicly funded biobank. The remaining participants were split, rating compensation as either moderately (24.9%) or rather/very important (18.3%). The more important a respondent rated financial compensation, the less willing this person was to report hypothetical willingness to participate in a biobank (aRP: rather not important: 1.24, 95% CI: 1.14 – 1.34; very important: 0.79, 95% CI: 0.67 – 0.92).

Preferences on data ownership

Nearly half of all respondents indicated that they personally should own the data and samples donated to a biobank (49.5%, 95% CI: 47.8% – 51.2%). Other potential 'data owners' included:

For peer review only - http://bmjopen.bmj.com/site/about/guidelines.xhtml

universities involved with the biobank (11.0%, 95% CI: 10.0% - 12.1%), the biobank (10.7%, 95% CI: 9.7% - 11.8%), the specific researchers who make discoveries (9.5%, 95% CI: 8.5% - 10.5%) or the Swiss government (5.4%, 95% CI: 4.7% - 6.3%). Participants who felt that ownership should go to the biobank (aRP: 1.32, 95% CI: 1.21 – 1.45), the Swiss government (aRP: 1.23, 95% CI: 1.08 – 1.39), the universities involved with the biobank (aRP: 1.36, 95% CI: 1.24 – 1.48) and the specific researchers who make discoveries (aRP: 1.31, 95% CI: 1.18 – 1.45) were significantly more likely to be willing to hypothetically participate in a biobank compared to persons who preferred that data ownership belongs to them personally. Those that endorsed ownership by ‘no one’ (6.2%, 95% CI: 5.4% - 7.1%) were less likely to express willingness to participate (aRP: 0.76, 95% CI: 0.63 – 0.92).

Views on responsibilities for data management and storage

Concerning data management and storage, most respondents preferred governance by the biobank (i.e. the management board, 34.3%, 95% CI: 32.7% - 35.9%) or an independent expert committee (e.g. independent researchers: scientists and clinicians not associated with the biobank, 30.7%, 95% CI: 29.1% - 32.3%). Those who favoured an independent committee representing the public (e.g. citizens, patients, the public) (8.0%) were less willing to participate in a hypothetical biobank (aRP: 0.78, 95% CI: 0.67 – 0.91) compared to respondents preferring governance by the biobank.

Trusted actors

Figure 4 shows to what extent respondents trusted different actors to keep their data and samples confidential and protected if they had access to them in a biobank. Most respondents reported the strongest trust in their own doctor (76.3%), medical doctors in general (41.9%) and researchers at a university (41.7%). Health insurers (8.4%), pharmaceutical companies (6.4%), other for-profit companies from Switzerland (3.4%) and other global for-profit companies (2.0%) are trusted least.

Reported trust in researchers differed by levels of education. Those with tertiary education reported stronger trust in researchers at a university (aRP: 1.26, 95% CI: 1.15 – 1.39) or researchers at other public institutes (aRP: 1.53, 95% CI: 1.34 – 1.75) than did those with an upper secondary education. Trust in health insurers differed by language region, education, religion, and health status: Respondents living in the Italian speaking part (aRP: 0.56, 95% CI: 0.42 – 0.75) of Switzerland trusted health insurances significantly less than respondents living in the German-speaking part. Trust in health insurers decreased as educational level increased

For peer review only - http://bmjopen.bmj.com/site/about/guidelines.xhtml

(compulsory education or less: aRP: 1.51, 95% CI: 1.07 – 2.14; tertiary education: aRP: 0.67, 95% CI: 0.47 – 0.94), whereas it increased as self-reported religiosity decreased (somewhat religious: aRP: 0.68, 95% CI: 0.51 – 0.92; not at all religious: aRP: 0.45, 95% CI: 0.32 – 0.63). Finally, healthier respondents' trust in health insurers was lower than for persons reporting a very unhealthy health status (somewhat healthy: aRP: 0.33, 95% CI: 0.14 – 0.75; very healthy: aRP: 0.40, 95% CI: 0.17 – 0.92).

Strong trust in eight out of the nine mentioned institutions (see Figure 4) increased the willingness to participate in a hypothetical personalised health research study or biobank. Only trust in global private, for-profit companies did not influence the willingness to participate in such research studies. Respondents also distinguished whether for-profit companies are Swiss or not: respondents with strong trust in Swiss for-profit companies are more willing to participate in personalised health research (aRP: 1.33, 95% CI: 1.18 – 1.51) compared to respondents having low trust in these companies. Those with compulsory education or less were more likely to trust Swiss for-profit companies (aRP: 2.29, 95% CI: 1.35 – 3.85) than those with secondary or tertiary education.

Figure 4 around here

DISCUSSION

The survey assessed the preferences of the Swiss public regarding the management and governance of health data and biological samples in biobanks for personalised health research. We found that a majority of respondents hypothetically prefer anonymous or coded data storage in a biobank (52% and 43% respectively), yet also prefer to be re-contacted for every new project to use their data (39%) or state that their preference depends on the type of project being considered (29%). Material compensation is not important to provide their data and samples to a Swiss publicly funded biobank, but nearly 50% prefer to own their data personally. Respondents preferred biobanks (34%) and independent expert committees (31%) to be responsible for biobank governance. And finally, Swiss most trust their own doctor, doctors in general and researchers at universities to keep data confidential and protected, while for-profit companies (both global and Swiss), pharmaceutical companies and health insurances are not highly trusted.

These results highlight that the Swiss public wants to be in charge of how their data and samples are used, based on their preferences for study-specific consent and personal ownership of their data or samples. Switzerland strongly emphasizes individual autonomy and citizen participation

For peer review only

[18], with direct democracy being a core feature of the Swiss political system. Therefore, these preferences are consistent with traditional cultural perceptions about individual control. However, as providing study-specific consent is often technically demanding and resource-intensive, most Swiss university hospitals have introduced broad consent ("general consent" in Switzerland) [19]. They ask patients once for their consent to use their anonymised or coded data and samples for future, unspecified studies.

Across the globe, consent preferences for biobank research have been well studied, with divergent results. Whereas some studies of U.S. biobanks confirm that certain types of consent increase willingness to participate in research [20], others do not confirm this finding [21–23]. Most studies, however, identify a preference for broad consent [10], broad or study-specific consent [24] or finding both types of consent unacceptable [4,7]. In contrast, a pan-European study found that 67% prefer study-specific consent, with only 24% opting for broad consent [25]. Public preferences depend on context and culture. They are influenced by how questions are framed, which population is included (the general population, research participants, patients), the country where the study takes place and its cultural values and norms. Country-specific variation of preferences is, therefore, expected. Our finding that most Swiss respondents preferred study-specific informed consent is consistent with previously published European data [25]. It reflects the above-mentioned cultural preference to maintain a level of control when deciding for which studies their data can be used [20]. It is noteworthy that existing literature about types of consent and data management is mostly U.S. based, highlighting the importance of studying public preferences for biobank management and governance in other countries.

Another important finding is that Swiss respondents who preferred their data to be stored anonymously also frequently indicated that they want to be re-contacted for every new project (34.9%). Re-contacting participants when their data is stored anonymously requires enormous effort. The conflicting preferences for anonymous data storage and the wish to control data use highlight that the public might lack sufficient knowledge and insights about data handling processes. Our survey asked about consent and storage preferences in two different, unlinked questions (first about consent, then about storage). Therefore, we do not have insights about the trade-off the public would make when choosing between having their data stored anonymously or being able to re-consent to each study using their data or samples. Future research should assess the public's specific preferences. However, re-consent rules many years after original consent will likely result in selection bias. For example, some participants will have died and will no longer be able to re-consent.

For peer review only

Respondents in our study trust health professionals and institutions they know, namely their own doctors, universities and Swiss companies. They expressed the least trust in pharmaceutical companies, global for-profit companies and health insurance companies. Other studies assessing public opinions globally [26] and in the U.K., U.S., Australia, Canada and Singapore [2,27–32] confirm that people distinguish between research conducted by public and private actors. Our study found another distinction regarding for-profit companies: Strong trust in Swiss for-profit companies increased the willingness to participate in personalised health research. In contrast, trust in global for-profit companies does not influence the willingness to participate. Recent international scandals on data sharing by global for-profit companies, such as the 'Project Nightingale' scandal, where a privately-held hospital chain shared medical records with Google without informing the data subjects [33], might have caused distrust in such actors. A similar debate occurred in summer 2021 when the British NHS planned to collect, pool and share patient data held by general practitioners as part of the so-called General Practice Data for Planning and Research (GPDPR) [34]. The initiative was set up as an automatic opt-in process with a six-week opt-out window and no option to delete data retrospectively. It included medical record data, information on sex, ethnicity, postal code, and week and year of birth (making data easily re-identifiable). Data was to be shared with health care and research organisations and commercial entities. Soon after its announcement, it was widely criticised as a "complete failure to develop a wide-ranging and far-reaching public engagement plan to communicate with the population" and put on hold [34]. NHS Digital then initiated a consultation process and a public information campaign to precede implementation. This experience highlights that engaging with the public and ensuring opportunities to make informed choices about their data is crucial for sustainable and trusted health data use.

Given these insights, we might ask: What should Swiss biobanks do to meet the public's needs and expectations better and increase trust? A first step to achieve this is for biobanks to increase transparency about governance mechanisms and information about research with health data and samples. We found that respondents unwilling to provide data or samples for research were also unsure about their preferred type of consent (80%) and were more likely to prefer that data be stored anonymously (58%). A lack of familiarity about how data and samples can be used for research purposes might thus explain disinterest in participation. Therefore, biobanks and other institutions collecting health data and samples should provide accessible and understandable information via websites and brochures about how data can be reused and the advantages and disadvantages of different types of consent for both the individual and society. This would also help educate about the types of data storage, e.g. anonymous vs. coded data

For peer review only - http://bmjopen.bmj.com/site/about/guidelines.xhtml

and how the data type influences the ability to be re-contacted or receive individual research results. Communication about future studies would also promote the participants' knowledge about the use of their data and samples and could help address perceived uncertainty that arises with data donation and reuse, and allow participants a sense of meaningful control over their data and samples [35]. To alleviate this, Switzerland may wish to further consider to develop dynamic consent models [16], which allow participants to personalise their consent preferences. Our data suggest that dynamic consent models better mirror the preferences of the Swiss public and permit participants to align future use of their data with their personal values. Such a move towards more granular, interactive consent models allows participants to retain a sense of increased control over their data and samples than is possible with broad consent only [35].

Next, strengthening trust in biobanks and research initiatives will require increased transparency about with whom and how data is shared for research use. Concerning the planned research, biobanks should specify for which projects data and samples will be used to any degree known and be transparent about the range of future potential research that may occur. This should also include whether they may share data with the private sector, such as pharmaceutical or medical technology companies [31]. In addition to listing all actors who have access to public data, the reasons and details of the cooperation should also be made transparent. In this way, potential data donors can decide for themselves whether they agree to such potential use of their data or samples before giving their consent.

As a way to increase trust, biobanks should treat the decision to donate data and samples as a process rather than a one-time signature of the consent form [7]. Given that respondents in our study and other studies [27,30] frequently trust their own doctors most, biobanks and research initiatives should consider that doctors could play a primary role in informing patients about donating data or samples [36]. Shifting the responsibilities and first point of contact to primary care would have the additional advantage of broader awareness. Public trust is a dynamic construct [37,38]. It can be easily weakened if biobanks and research institutions do not meet expectations that originally made them trustworthy from the public perspective [39,40]. The systemic oversight approach can yield helpful mechanisms to build and maintain public trust in increasingly complex research initiatives [35]. It requires that oversight mechanisms are adaptive to treat different data sources and flexible enough to assess their intended use. Dynamic monitoring should take place that is responsive to containing risks for data subjects.

As previously described [3], a limitation of our study is that this survey of Swiss residents only achieved 34% response rate. However, as we had the full sampling list, we could construct non-response weights by all sociodemographic characteristics (age, sex, regions, and languages).

For peer review only

Such weighting, therefore, reduces the possible non-response bias, making the results reasonably generalisable to the Swiss population. For this report, we collapsed a 5 point Likert scale for levels of trust (1 = no trust to 5 = strong trust) to binary variables (low trust, strong trust). This approach will have removed nuances of respondents' attitudes towards different actors. And finally, as previously noted, we also asked about the type of consent and storage preferences in two separate questions, making it impossible to determine which one is more important to our respondents.

CONCLUSIONS

This study reveals what the Swiss public expects from data management and governance when donating health data or biological samples to personalised health research. This study aimed to close the research gap on the Swiss public's preferences on data storage, consent, ownership, and management responsibilities while also measuring trust levels in different health care actors to keep data and samples confidential and protected. These insights into the public's preferences on data management and governance should allow Swiss biobanks and research institutions to consider the expectations and preferences of potential data donors. Aligning governance strategies with public expectations not only promotes trust in biobank endeavours [41], the resulting trust also positively influences the public's willingness to participate in health research and biobanks [42]. Transparent communication about the research, uses of data, and implementation of consent models where participants can choose how often they would like to be asked for permission to use their data (dynamic consent) are key to increasing Swiss individuals' willingness to provide their data to biobanks.

For peer review only

Ethics approval: The Ethics Committee of ETH Zurich (EK 2018-N-66) approved the study.

Acknowledgments: We thank members at the Health Ethics & Policy Lab at ETH Zurich for discussions on the questionnaire development and pre-testing, Christoph Freymond from the Federal Office for Statistics for providing the data sample, Stefan Wehrli and his team from the Decision Sciences Laboratory at ETH Zurich for setting up and maintaining the Call Center and data collection infrastructure and Jan Kaiser for automating the digitalisation of the paper-based questionnaires. We especially thank the respondents who took part in the survey.

Competing interests: None declared.

Funding: This work was supported by Swiss National Science Foundation (SNSF) Grant 157556 to E.V. and through the personal research fund of E.V. at ETH Zurich, which covered the costs of data collection. M.E. was supported by special project funding (Grant 189498) from the SNSF.

Author Contributions: Conceptualization, C.Be., M.Z., M.E., E.V.; methodology, C.Br., C.Be., M.Z., E.V., M.E., K.E.O.; software, C.Be.; validation, C.Br., C.Be., M.Z., K.E.O.; formal analysis, C.Br., C.Be., M.Z.; investigation, C.Br., C.Be., M.Z., E.V., M.E., K.E.O.; resources, E.V.; data curation, C.Br., C.Be.; writing—original draft preparation, C.Br.; writing—review and editing, C.Br., C.Be., M.Z., E.V., M.E., K.E.O.; visualization, C.Br., C.Be.; supervision, M.Z., E.V., K.E.O.; project administration, C.Br.; funding acquisition, M.E., E.V. All authors have read and agreed to the published version of the manuscript.

Data Sharing Statement: Dataset available from ETH Zurich, DOI: 10.3929/ethz-b-000474690).

For peer review only

REFERENCES

1 Flores M, Glusman G, Brogaard K, *et al.* P4 medicine: How systems medicine will transform the healthcare sector and society. *Per Med* 2013;**10**:565–576. doi:10.2217/pme.13.57

2 Vidgen ME, Kaladharan S, Malacova E, *et al.* Sharing genomic data from clinical testing with researchers: public survey of expectations of clinical genomic data management in Queensland, Australia. *BMC Med Ethics* 2020;**21**:1–11. doi:10.1186/s12910-020-00563-6

3 Brall C, Berlin C, Zwahlen M, *et al.* Public willingness to participate in personalized health research and biobanking: A large-scale Swiss survey. *PLoS One* 2021;**16**:e0249141. doi:10.1371/journal.pone.0249141

4 Tomlinson T, De Vries R, Ryan K, *et al.* Moral concerns and the willingness to donate to a research biobank. *JAMA - J Am Med Assoc* 2015;**313**:417–9. doi:10.1001/jama.2014.16363

5 Clayton EW, Halverson CM, Sathe NA, *et al.* A systematic literature review of individuals' perspectives on privacy and genetic information in the United States. *PLoS One* 2018;**13**:1–26. doi:10.1371/journal.pone.0204417

6 Goddard KAB, Smith KS, Chen C, *et al.* Biobank Recruitment: Motivations for Nonparticipation. *Biopreserv Biobank* 2009;**7**:119–21. doi:10.1089/bio.2009.0006

7 De Vries RG, Tomlinson T, Kim HM, *et al.* Understanding the public's reservations about broad consent and study-by-study consent for donations to a biobank: Results of a national survey. *PLoS One* 2016;**11**:1–11. doi:10.1371/journal.pone.0159113

8 D'Abramo F, Schildmann J, Vollmann J. Research participants' perceptions and views on consent for biobank research: a review of empirical data and ethical analysis. *BMC Med Ethics* 2015;**16**:1–11. doi:10.1186/s12910-015-0053-5

9 Smith ME, Sanderson SC, Brothers KB, *et al.* Conducting a large, multi-site survey about patients' views on broad consent: challenges and solutions. *BMC Med Res Methodol* 2016;**16**:1–11. doi:10.1186/s12874-016-0263-7

10 Garrison NA, Sathe NA, Antommaria AHM, *et al.* A systematic literature review of individuals' perspectives on broad consent and data sharing in the United States. *Genet Med* 2016;**18**:663–71. doi:10.1038/gim.2015.138

11 Grady C, Eckstein L, Berkman B, *et al.* Broad Consent for Research With Biological Samples: Workshop Conclusions. *Am J Bioeth* 2015;**15**:34–42. doi:10.1080/15265161.2015.1062162

For peer review only - http://bmjopen.bmj.com/site/about/guidelines.xhtml

12 Dankar FK, Gergely M, Dankar SK. Informed Consent in Biomedical Research. *Comput Struct Biotechnol J* 2019;**17**:463–74. doi:10.1016/j.csbj.2019.03.010

13 Wendler D. Broad versus Blanket Consent for Research with Human Biological Samples. *Hastings Cent Rep* 2013;**43**:3–4. doi:10.1002/hast.200

14 Blasimme A, Brall C, Vayena E. Reporting Genetic Findings to Individual Research Participants: Guidelines From the Swiss Personalized Health Network. *Front Genet* 2020;**11**. doi:10.3389/fgene.2020.585820

15 Scheibner J, Ienca M, Kechagia S, *et al.* Data protection and ethics requirements for multisite research with health data: A comparative examination of legislative governance frameworks and the role of data protection technologies. *J Law Biosci* 2020;**7**:1–30. doi:10.1093/jlb/lsaa010

16 Meier-Abt P, Lawrence AK, Selter L, *et al.* The Swiss approach to precision medicine. *Swiss Med Wkly* 2018;:1–8.

17 Zou G. A Modified Poisson Regression Approach to Prospective Studies with Binary Data. *Am J Epidemiol* 2004;**159**:702–6. doi:10.1093/aje/kwh090

18 Biller-Andorno N, Zeltner T. Individual Responsibility and Community Solidarity — The Swiss Health Care System. *N Engl J Med* 2015;**373**:2193–7. doi:10.1056/NEJMp1508256

19 Universitäre Medizin Schweiz. Generalkonsent für die Forschung. https://www.unimedsuisse.ch/de/projekte/generalkonsent (accessed 9 May 2021).

20 Goodman D, Bowen D, Wenzel L, *et al.* The Research participant perspective related to the conduct of genomic cohort studies: A systematic review of the quantitative literature. *Transl Behav Med* 2018;**8**:119–29. doi:10.1093/tbm/ibx056

21 Kaufman DJ, Baker R, Milner LC, *et al.* A survey of U.S adults' opinions about conduct of a nationwide Precision Medicine Initiative® cohort study of genes and environment. *PLoS One* 2016;**11**:1–14. doi:10.1371/journal.pone.0160461

22 Sanderson SC, Brothers KB, Mercaldo ND, *et al.* Public Attitudes toward Consent and Data Sharing in Biobank Research: A Large Multi-site Experimental Survey in the US. *Am J Hum Genet* 2017;**100**:414–27. doi:10.1016/j.ajhg.2017.01.021

23 Gayet-Ageron A, Rudaz S, Perneger T. Biobank attributes associated with higher patient participation: A randomized study. *Eur J Hum Genet* 2016;**25**:31–6. doi:10.1038/ejhg.2016.132

24 Ewing AT, Erby LAH, Bollinger J, *et al.* Demographic differences in willingness to provide broad and narrow consent for biobank research. *Biopreserv Biobank*

For peer review only - http://bmjopen.bmj.com/site/about/guidelines.xhtml

2015;**13**:98–106. doi:10.1089/bio.2014.0032

25 Gaskell G, Gottweis H, Starkbaum J, *et al.* Publics and biobanks: Pan-European diversity and the challenge of responsible innovation. *Eur J Hum Genet* 2013;**21**:14–20. doi:10.1038/ejhg.2012.104

26 Middleton A, Milne R, Almarri MA, *et al.* Global Public Perceptions of Genomic Data Sharing: What Shapes the Willingness to Donate DNA and Health Data? *Am J Hum Genet* 2020;**107**:743–52. doi:10.1016/j.ajhg.2020.08.023

27 Ghafur S, Van Dael J, Leis M, *et al.* Public perceptions on data sharing: key insights from the UK and the USA. *Lancet Digit Heal* 2020;**2**:e444–6. doi:10.1016/S2589-7500(20)30161-8

28 IPSOS MORI for Wellcome Trust. The One-Way Mirror: Public attitudes to commercial access to health data Report prepared for the Wellcome Trust. 2016.

29 Trinidad SB, Fullerton SM, Bares JM, *et al.* Genomic research and wide data sharing: Views of prospective participants. *Genet Med* 2010;**12**:486–95. doi:10.1097/GIM.0b013e3181e38f9e

30 Milne R, Morley KI, Howard H, *et al.* Trust in genomic data sharing among members of the general public in the UK, USA, Canada and Australia. *Hum Genet* 2019;**138**:1237–46. doi:10.1007/s00439-019-02062-0

31 Braunack-Mayer A, Fabrianesi B, Street J, *et al.* Sharing Government Health Data With the Private Sector: Community Attitudes Survey. *J Med Internet Res* 2021;**23**:e24200. doi:10.2196/24200

32 Lysaght T, Ballantyne A, Toh HJ, *et al.* Trust and Trade-Offs in Sharing Data for Precision Medicine: A National Survey of Singapore. *J Pers Med* 2021;**11**:921. doi:10.3390/jpm11090921

33 Copeland R. Google's 'Project Nightingale' Gathers Personal Health Data on Millions of Americans. Wall Str. J. 2019.https://www.wsj.com/articles/google-s-secret-project-nightingale-gathers-personal-health-data-on-millions-of-americans-11573496790 (accessed 7 Jun 2021).

34 Burki T. Concerns over England's new system for collecting general practitioner data. *Lancet Digit Heal* 2021;**3**:e469–70. doi:10.1016/S2589-7500(21)00148-5

35 Vayena E, Blasimme A. Health research with big data: Time for systemic oversight. *J Law, Med Ethics* 2018;**46**:119–29. doi:10.1177/1073110518766026

36 Kelly SE, Spector TD, Cherkas LF, *et al.* Evaluating the consent preferences of UK research volunteers for genetic and clinical studies. *PLoS One* 2015;**10**:1–12.

For peer review only

doi:10.1371/journal.pone.0118027

37 Gille F, Brall C. Can we know if donor trust expires? About trust relationships and time in the context of open consent for future data use. *J Med Ethics* 2021;:medethics-2020-106244. doi:10.1136/medethics-2020-106244

38 Kerasidou A. The role of trust in global health research collaborations. *Bioethics* 2019;**33**:495–501. doi:10.1111/bioe.12536

39 Shabani M, Bezuidenhout L, Borry P. Attitudes of research participants and the general public towards genomic data sharing: A systematic literature review. *Expert Rev Mol Diagn* 2014;**14**:1053–65. doi:10.1586/14737159.2014.961917

40 Gille F, Axler R, Blasimme A. Transparency about Governance Contributes to Biobanks' Trustworthiness: Call for Action. *Biopreserv Biobank* 2021;**19**:83–5. doi:10.1089/bio.2020.0057

41 Dive L, Critchley C, Otlowski M, *et al.* Public trust and global biobank networks. *BMC Med Ethics* 2020;**21**:1–9. doi:10.1186/s12910-020-00515-0

42 Middleton A, Milne R, Almarri MA, *et al.* Global Public Perceptions of Genomic Data Sharing: What Shapes the Willingness to Donate DNA and Health Data? *Am J Hum Genet* 2020;**107**:1–10. doi:10.1016/j.ajhg.2020.08.023

For peer review only

Legends

Figure 1. Overview of how often the biobank should ask for permission to re-use the data (type of consent). (**a**) Overall percentages of respondents who preferred various options regarding how often the biobank should ask for permission to re-use the data (type of consent). “Ask me only once” corresponds to a ‘broad consent’ approach, and “Ask me again for every new project” corresponds to specific consent and/or a dynamic consent approach. (**b**) Percent of each response choice who are willing / not willing to participate in personalized health research.

Figure 2. Preferences on data and sample storage. (**a**) Percentages of respondents who preferred each approach to data and sample storage; (**b**) Percent of each response choice who are willing / not willing to participate in personalized health research.

Figure 3. Preferences for information necessary in order to make a decision to donate data and samples to a biobank. Figure presents population weighted percentages; respondents were asked to indicate their top three informational preferences. * indicates statistical difference between the two groups of respondents willing and not willing to participate, $p<0.05$.

Figure 4. Trust in actors to keep data protected. Figure presents population weighted percentages of total respondents.

For peer review only

For peer review only - http://bmjopen.bmj.com/site/about/guidelines.xhtml

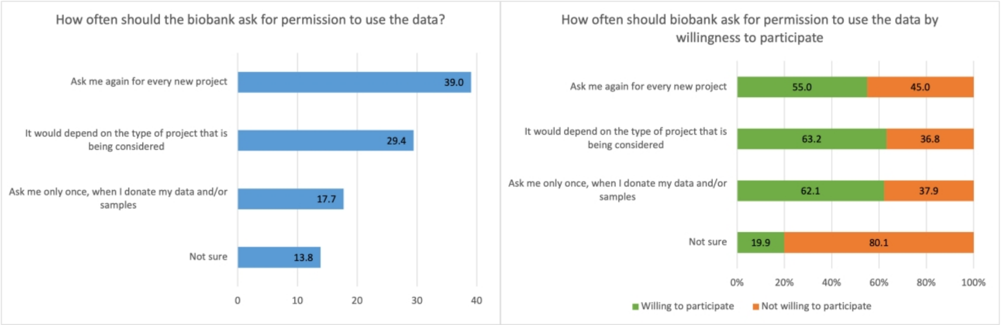

Figure 1. Overview of how often the biobank should ask for permission to re-use the data (type of consent). (a) Overall percentages of respondents who preferred various options regarding how often the biobank should ask for permission to re-use the data (type of consent). “Ask me only once” corresponds to a ‘broad consent’ approach, and “Ask me again for every new project” corresponds to specific consent and/or a dynamic consent approach. (b) Percent of each response choice who are willing / not willing to participate in personalized health research.

290x94mm (300 x 300 DPI)

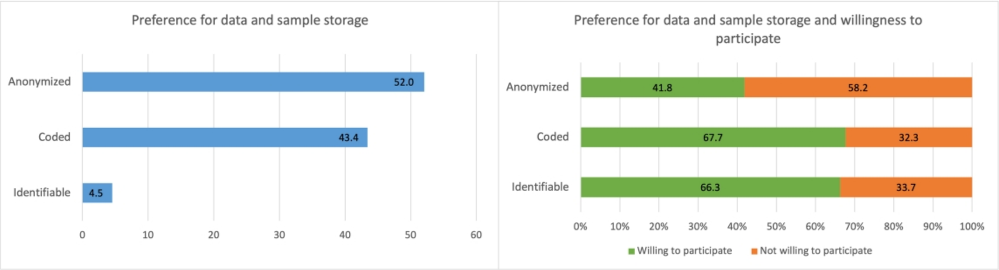

Figure 2. Preferences on data and sample storage. (a) Percentages of respondents who preferred each approach to data and sample storage; (b) Percent of each response choice who are willing / not willing to participate in personalized health research.

284x76mm (300 x 300 DPI)

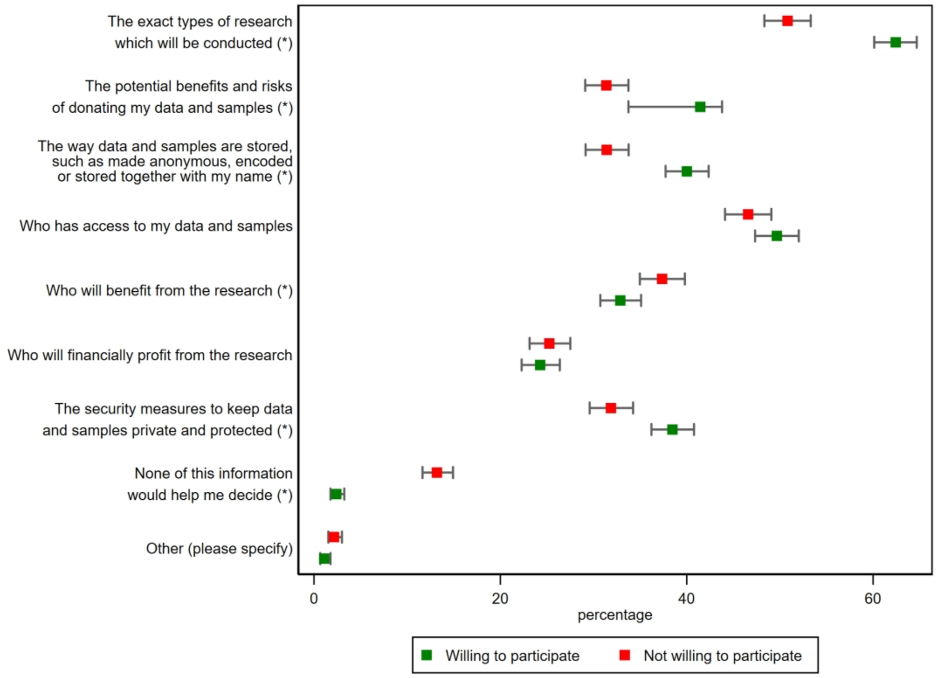

Figure 3. Preferences for information necessary in order to make a decision to donate data and samples to a biobank. Figure presents population weighted percentages; respondents were asked to indicate their top three informational preferences. * indicates statistical difference between the two groups of respondents willing and not willing to participate, p<0.05.

705x513mm (72 x 72 DPI)

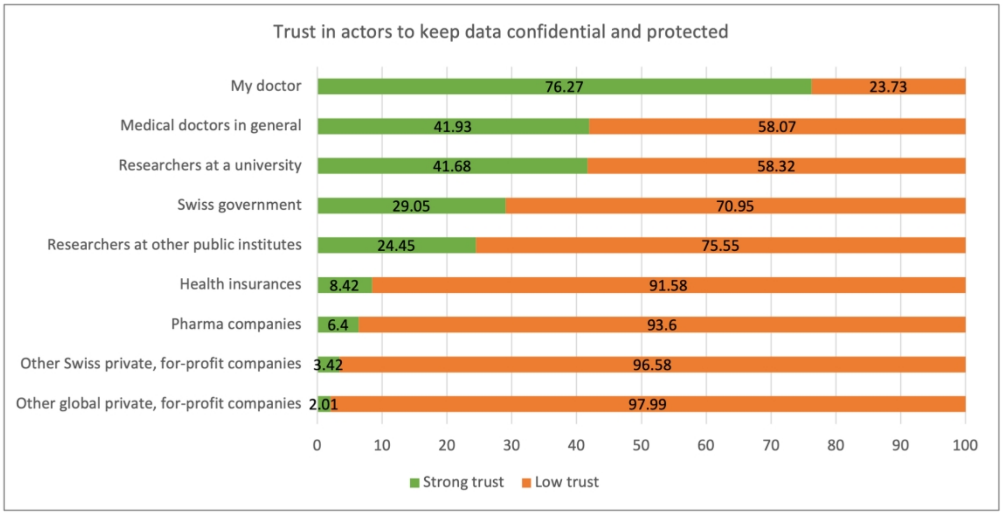

Figure 4. Trust in actors to keep data protected. Figure presents population weighted percentages of total respondents.

188x96mm (300 x 300 DPI)

STROBE Statement—Checklist of items that should be included in reports of ***cross-sectional studies***

	Item No	Recommendation	Page No
**Title and abstract**	1	(*a*) Indicate the study's design with a commonly used term in the title or the abstract	1
		(*b*) Provide in the abstract an informative and balanced summary of what was done and what was found	2
**Introduction**			
Background/rationale	2	Explain the scientific background and rationale for the investigation being reported	3-4
Objectives	3	State specific objectives, including any prespecified hypotheses	4
**Methods**			
Study design	4	Present key elements of study design early in the paper	4
Setting	5	Describe the setting, locations, and relevant dates, including periods of recruitment, exposure, follow-up, and data collection	4-5
Participants	6	(*a*) Give the eligibility criteria, and the sources and methods of selection of participants	4
Variables	7	Clearly define all outcomes, exposures, predictors, potential confounders, and effect modifiers. Give diagnostic criteria, if applicable	5
Data sources/ measurement	8*	For each variable of interest, give sources of data and details of methods of assessment (measurement). Describe comparability of assessment methods if there is more than one group	5
Bias	9	Describe any efforts to address potential sources of bias	5
Study size	10	Explain how the study size was arrived at	4
Quantitative variables	11	Explain how quantitative variables were handled in the analyses. If applicable, describe which groupings were chosen and why	5
Statistical methods	12	(*a*) Describe all statistical methods, including those used to control for confounding	5
		(*b*) Describe any methods used to examine subgroups and interactions	5
		(*c*) Explain how missing data were addressed	5
		(*d*) If applicable, describe analytical methods taking account of sampling strategy	5
		(*e*) Describe any sensitivity analyses	5
**Results**			
Participants	13*	(a) Report numbers of individuals at each stage of study—eg numbers potentially eligible, examined for eligibility, confirmed eligible, included in the study, completing follow-up, and analysed	4, reported elsewhere (Brall et al., 2021)
		(b) Give reasons for non-participation at each stage	4, reported elsewhere (Brall et al., 2021)

For peer review only - http://bmjopen.bmj.com/site/about/guidelines.xhtml

		(c) Consider use of a flow diagram	/
Descriptive data	14*	(a) Give characteristics of study participants (eg demographic, clinical, social) and information on exposures and potential confounders	6-10
		(b) Indicate number of participants with missing data for each variable of interest	4, reported elsewhere (Brall et al., 2021)
Outcome data	15*	Report numbers of outcome events or summary measures	6-13
Main results	16	(*a*) Give unadjusted estimates and, if applicable, confounder-adjusted estimates and their precision (eg, 95% confidence interval). Make clear which confounders were adjusted for and why they were included	6-13
		(*b*) Report category boundaries when continuous variables were categorized	6-13
		(*c*) If relevant, consider translating estimates of relative risk into absolute risk for a meaningful time period	/
Other analyses	17	Report other analyses done—eg analyses of subgroups and interactions, and sensitivity analyses	6-13
**Discussion**			
Key results	18	Summarise key results with reference to study objectives	13-14
Limitations	19	Discuss limitations of the study, taking into account sources of potential bias or imprecision. Discuss both direction and magnitude of any potential bias	17
Interpretation	20	Give a cautious overall interpretation of results considering objectives, limitations, multiplicity of analyses, results from similar studies, and other relevant evidence	14-16
Generalisability	21	Discuss the generalisability (external validity) of the study results	17
**Other information**			
Funding	22	Give the source of funding and the role of the funders for the present study and, if applicable, for the original study on which the present article is based	18

*Give information separately for exposed and unexposed groups.

Note: An Explanation and Elaboration article discusses each checklist item and gives methodological background and published examples of transparent reporting. The STROBE checklist is best used in conjunction with this article (freely available on the Web sites of PLoS Medicine at http://www.plosmedicine.org/, Annals of Internal Medicine at http://www.annals.org/, and Epidemiology at http://www.epidem.com/). Information on the STROBE Initiative is available at www.strobe-statement.org.

For peer review only

BMJ Open

Public preferences towards data management and governance in Swiss biobanks – results from a nationwide survey

Journal:	*BMJ Open*
Manuscript ID	bmjopen-2022-060844.R1
Article Type:	Original research
Date Submitted by the Author:	16-Jun-2022
Complete List of Authors:	Brall, Caroline; ETH Zurich, Department of Health Sciences and Technology<br>Berlin, Claudia; University of Bern, Institute of Social and Preventive Medicine<br>Zwahlen , Marcel; University of Bern, Insitute of Social and Preventive Medicine<br>Vayena, Effy; ETH Zurich, Department of Health Sciences and Technology<br>Egger, Matthias; University of Bern, Institute of Social and Preventive Medicine, Bristol Medical School; University of Bristol, Population Health Sciences<br>Ormond, Kelly E.; ETH Zurich, Department of Health Sciences and Technology
**Primary Subject Heading**:	Health policy
Secondary Subject Heading:	Genetics and genomics, Health services research, Medical management
Keywords:	PUBLIC HEALTH, Health policy < HEALTH SERVICES ADMINISTRATION & MANAGEMENT, Information management < BIOTECHNOLOGY & BIOINFORMATICS

SCHOLARONE™
Manuscripts

I, the Submitting Author has the right to grant and does grant on behalf of all authors of the Work (as defined in the below author licence), an exclusive licence and/or a non-exclusive licence for contributions from authors who are: i) UK Crown employees; ii) where BMJ has agreed a CC-BY licence shall apply, and/or iii) in accordance with the terms applicable for US Federal Government officers or employees acting as part of their official duties; on a worldwide, perpetual, irrevocable, royalty-free basis to BMJ Publishing Group Ltd ("BMJ") its licensees and where the relevant Journal is co-owned by BMJ to the co-owners of the Journal, to publish the Work in this journal and any other BMJ products and to exploit all rights, as set out in our licence.

The Submitting Author accepts and understands that any supply made under these terms is made by BMJ to the Submitting Author unless you are acting as an employee on behalf of your employer or a postgraduate student of an affiliated institution which is paying any applicable article publishing charge ("APC") for Open Access articles. Where the Submitting Author wishes to make the Work available on an Open Access basis (and intends to pay the relevant APC), the terms of reuse of such Open Access shall be governed by a Creative Commons licence – details of these licences and which Creative Commons licence will apply to this Work are set out in our licence referred to above.

Other than as permitted in any relevant BMJ Author's Self Archiving Policies, I confirm this Work has not been accepted for publication elsewhere, is not being considered for publication elsewhere and does not duplicate material already published. I confirm all authors consent to publication of this Work and authorise the granting of this licence.

For peer review only

Public preferences towards data management and governance in Swiss biobanks – results from a nationwide survey

Caroline Brall[1], Claudia Berlin[2], Marcel Zwahlen[2], Effy Vayena[1], Matthias Egger[2,3] and Kelly E. Ormond[1]

[1] Health Ethics and Policy Lab, Department of Health Sciences and Technology, ETH Zurich, Zurich, Switzerland
[2] Institute of Social and Preventive Medicine, University of Bern, Bern, Switzerland
[3] Population Health Sciences, Bristol Medical School, University of Bristol, Bristol, UK

Corresponding author: Caroline Brall, Hottingerstrasse 10, 8092 Zurich, carobrall@gmail.com

Word count: 4567

For peer review only

Abstract

Objectives: This article aims to measure the willingness of the Swiss public to participate in personalised health research, and their preferences regarding data management and governance.

Setting: Results are presented from a nationwide survey of members of the Swiss public.

Participants: 15,106 randomly selected Swiss residents received the survey in September 2019. The response rate was 34.1% (n = 5,156). Respondent age ranged from 18 to 79 years, with fairly uniform spread across sex and age categories between 25 – 64 years.

Primary and secondary outcome measures: Willingness to participate in personalised health research and opinions regarding data management and governance.

Results: Most respondents preferred to be contacted and re-consented for each new project using their data (39%, 95% CI: 37.4% - 40.7%), or stated that their preference depends on the project type (29.4%, 95% CI: 27.9% - 31%). Additionally, a majority (52%, 95% CI: 50.3% - 53.8%) preferred their data or samples be stored anonymously or in coded form (43.4%, 95% CI: 41.7% - 45.1%). Of those who preferred that their data be anonymised, most also indicated a wish to be re-contacted for each new project (36.8%, 95% CI: 34.5% - 39.2%); however these preferences are in conflict. Most respondents desired to personally own their data. Finally, most Swiss respondents trust their doctors, along with researchers at universities, to protect their data.

Conclusion: Insight into public preference can enable Swiss biobanks and research institutions to create management and governance strategies that match the expectations and preferences of potential participants. Models allowing participants to choose how to interact with the process, while more complex, may increase individual willingness to provide data to biobanks.

For peer review only

Strengths and limitations of this study

- This nationwide study examined preferences of 5,156 members of the Swiss public regarding data management and governance when hypothetically providing data or samples for personalised health research.
- We were able to construct non-response weights by all sociodemographic characteristics, making the results reasonably generalisable to the Swiss population.
- Respondents were asked about type of consent and storage preferences in two separate questions, making it impossible to determine which one is more important to respondents.

For peer review only - http://bmjopen.bmj.com/site/about/guidelines.xhtml

Keywords: Public preferences, biobanks, data management, data governance, personalised health research, Switzerland

For peer review only

For peer review only - http://bmjopen.bmj.com/site/about/guidelines.xhtml

INTRODUCTION

Personalised health strives to improve clinical outcomes by providing more effective prevention and therapy for individuals, thus improving morbidity and mortality. Large amounts of health data and samples are necessary to achieve the goals of personalised medicine, requiring that individuals participate in biomedical research by donating their health data and samples, and that the samples and data be available for reuse in future research studies [1]. Studies have shown that public preferences around sharing personal health and genetic information in a research setting vary depending on societal, demographic and jurisdictional context [2]. In Switzerland, we previously found that the public generally holds a positive opinion towards personalised health research, and that a majority are hypothetically willing to donate data and samples for such projects [3]. Yet in biobank research, concerns about data privacy and reuse are closely linked to public willingness to participate in research [3–6].

Two research design components greatly influence the ability to reuse samples and data for future research, and participants' willingness to provide samples [7]. First, a biobank can choose to store data in an identifiable, coded or anonymous form. If data and samples are stored and labelled with identifiable information, they can easily be linked back to donors. In contrast, anonymous data storage removes all personal identifiers from the data; linking back to a specific person is possible, but only with tremendous effort. An intermediate option is storage in coded form, which links an individual's data and samples via a securely stored code. Second, the way in which informed consent occurs can influence participant trust and the feasibility of sample and data reuse [8,9]. Broad consent (also known as general consent) denotes that individuals consent to their data and samples being used in future unspecified research studies. Broad consent approaches increase data availability, thereby facilitating future research, but rely on potential participants' comfort with agreeing to take part in future research without knowing what areas it will address [10]. On the other hand, specific consent requires that individuals provide consent for every study that explicitly uses their data or samples. Other more specific consent models exist beyond those presented, including blanket consent, presumed consent with opt-out, or tiered consent (for a detailed description see [11–13]). While study-specific consent is often technically challenging and resource-intensive to implement and update, it allows participants greater control over their samples and data, particularly in the case of potentially stigmatising or controversial research. Finally, choices in the research design for data storage and consent approachinfluence how data can be used, whether individuals must or can be re-contacted for consent, and possibilities for reporting individual results [14].

For peer review only - http://bmjopen.bmj.com/site/about/guidelines.xhtml

In Switzerland, existing biobanks are primarily linked to five university hospitals and generally use a broad consent approach [15,16]. Personalised health initiatives are scaling up, and broad consent has been promoted at the national level [16]. However, we do not know how the Swiss public views this type of consent, and what other views and expectations they may hold concerning the management and governance of their health data and samples. A better understanding of what potential participants expect from personalised health biobanks and related research infrastructure, and the data management and governance practices that influence willingness to donate data and samples, will allow biobanks to address the needs of the public in the early stages of project development.

This paper presents findings from a nationwide survey [3] of the Swiss public's preferences for data management and governance when hypothetically providing data or samples for personalised health research. We report preferences for consent type, data storage, ownership and management responsibilities, and information respondents need before donating data or samples. Finally, we present respondents' levels of trust in various actors across the health data ecosystem.

METHODS

We conducted a cross-sectional survey, with full details of the survey development and pilot testing published previously [3]. Briefly, the survey contained questions to assess individual attitudes, concerns and expectations towards hypothetically providing health data or biological samples for personalised health research. It consisted of six parts: general attitudes; motivations and barriers to participating in personalised health research; expectations towards data management; data governance; data sharing and uses; and willingness to receive results. The questionnaire was available in English, German, Italian and French, and consisted of 23 closed (binary, 5-point Likert scale and multiple choice) questions. This paper will focus on the findings for data management, data governance and data sharing.

The survey was mailed to 15,106 Swiss residents over the age of 18 in September 2019. According to the Swiss Statistical Survey Ordinance, the Swiss Federal Statistical Office (FSO) provided the stratified random sample, which covered the three main language regions (SR 431.012.1, article 13c). The sampled potential participants received the survey by regular mail in their language of correspondence (indicated at their municipality). The survey could be completed either on paper or through a web link on the Qualtrics survey platform (Qualtrics, Provo, Utah 84604, USA); both options allowed mapping of individual responses with sociodemographic characteristics provided by the FSO. Participants received two reminders

For peer review only - http://bmjopen.bmj.com/site/about/guidelines.xhtml

(after three and seven weeks). Responses were collected over 20 weeks from September 2019 to January 2020. By responding to the survey, participants provided their informed consent. We complied with data protection regulations at both research institutions (ETH Zurich and the University of Bern) and obtained ethics approval from the Ethics Committee of ETH Zurich (EK 2018-N-66).

Public involvement

The development of the research question and outcome measures was informed by the aim to learn more about public preferences and expectations towards donating health data or samples for personalised health research. Seventeen members of the public were involved in testing preliminary versions of the questionnaire, which was adapted according to their comments.

As we indicated to respondents that they would not be contacted again after completing the survey, results were not actively disseminated to participants, but made available upon request. In addition, the results are published open access.

Statistical analysis

After evaluating the data for completeness (minimum of 50% data completeness for inclusion in analysis), we analysed it using STATA (version 15, College Station, TX, USA). We linked the survey data with demographic data from the FSO (gender, age, language, household size, nationality, marital status and municipality of residence) using the FSO unique identifiers. To account for differences between survey respondents and the general population of Switzerland, we applied survey weights using gender, age and language region, and included additional variables provided by the FSO. Our final dataset included age, gender, nationality, number of household members, marital status, having biological children, language region, type of municipality of residence (urban or rural), education, religiosity, current or previous employment in the health sector, health status, and type of survey response (online or paper-based). We excluded missing data and used relative proportions for statistical analysis, as they allow a more straightforward interpretation than odds ratios: comparing a proportion of 60% to one of 30% leads to a relative proportion of 2.0, but an odds ratio of 3.5. We used modified multivariable Poisson regression to analyse the relative proportion of respondents' willingness to provide health data or biological samples for personalised health research, and to adjust for the other respondent characteristics [17]. To simplify interpretation of the results, we collapsed a 5-point Likert scale (1 = no trust to 5 = strong trust) to binary variables (1-3 = low trust, 4-5 = strong trust).

For peer review only - http://bmjopen.bmj.com/site/about/guidelines.xhtml

RESULTS

Sample description

As reported in detail elsewhere [3], 5,086 complete responses were received, representing an overall response rate of 34.1%. Most responses (71.0%) were submitted via the web-based survey platform. Respondent age ranged from 18 to 79 years, with fairly even spread across sex and age categories between 25 – 64 years (Table 1). A majority of respondents were Swiss nationals (76.0%), lived in a German-speaking region (70.8%), in urban areas (61.0%), were married (50.9%), had children (57.6%) and lived in 3-5 person households (43.7%). Most had secondary education (65.2%) and described their health status as somewhat (47.1%) or very healthy (36.6%); a majority had never worked in the health sector (79.8%). Thirteen percent reported being very religious. More than half of respondents (53.6%) expressed a hypothetical willingness to provide health data or biological samples for personalised health research purposes [3].

Preferences on types of consent

Regarding consent to use data from a hypothetical biobank, 39.0% (95% CI: 37.4% - 40.7%) of respondents wished to be asked for consent for each new project (study-specific consent). Furthermore, 29.4% (95% CI: 27.9% - 31.0%) indicated that this would depend on the type of project, and 17.7% (95% CI: 16.4% - 19.0%) preferred to be asked only once when donating data or samples (general consent). A minority of respondents (13.8%, 95% CI: 12.7% - 15.1%) were unsure of their preference. Compared with those who wished to be asked only once, participants who wished to be asked for permission for each project or who were unsure were less willing to hypothetically provide data and samples for research (adjusted Relative Proportions (aRP) = 0.86, 95% CI: 0.79 - 0.93; aRP = 0.35, 95% CI: 0.29 - 0.43) (Figure 1). Table 1 shows demographic information for different types of consent. The proportion of those who were more inclined to be asked only once (general consent) varied for some naturally ordered characteristics but not for all when testing for a trend: age groups (p for trend <0.001), number of household members (p for trend =0.124), religiousness (p for trend =0.770), education (p for trend =0.003) and health status (p for trend =0.598).

For peer review only - http://bmjopen.bmj.com/site/about/guidelines.xhtml

Table 1. Preferences for consent type according to sociodemographic factors (all weighted proportions except N).

	Sample	Population	How often should a biobank ask for permission?			
			Ask me only once, when I donate my data or samples	Ask me again for every new project	It would depend on the type of project that is being considered	Not sure
	N	%	% [95% CI]	% [95% CI]	% [95% CI]	% [95% CI]
**Total**	**5,086**	**100**	**17.7 [16.4,19.0]**	**39.0 [37.4,40.7]**	**29.4 [27.9,31.0]**	**13.8 [12.7,15.1]**
**Age group**						
18 - 24	594	8.6	10.8 [8.2,14.1]	41.8 [37.0,46.7]	34.7 [30.1,39.6]	12.7 [9.8,16.4]
25 - 34	758	17.7	14.5 [11.7,17.9]	41.8 [37.6,46.2]	30.2 [26.3,34.4]	13.4 [10.7,16.7]
35 - 44	632	18.5	15.4 [12.3,19.1]	46.8 [42.2,51.5]	26.3 [22.4,30.6]	11.5 [8.9,14.8]
45 - 54	927	19.5	18.2 [15.4,21.4]	41.3 [37.6,45.1]	27.5 [24.2,31.1]	13.0 [10.6,15.9]
55 - 64	1,005	17.5	17.4 [14.8,20.4]	37.6 [34.0,41.4]	29.6 [26.3,33.2]	15.3 [12.8,18.3]
65 - 74	857	13.1	26.8 [23.3,30.5]	27.4 [24.0,31.1]	29.7 [26.2,33.4]	16.1 [13.5,19.2]
75 - 79	313	5.2	25.1 [19.7,31.5]	21.3 [16.3,27.2]	35.5 [29.3,42.2]	18.1 [13.5,23.8]
Total	5,086	100	17.7 [16.4,19.0]	39.0 [37.4,40.7]	29.4 [27.9,31.0]	13.8 [12.7,15.1]
**Sex**						
Male	2,451	50.1	18.8 [17.0,20.8]	38.3 [35.9,40.8]	29.5 [27.3,31.8]	13.4 [11.8,15.2]
Female	2,635	49.9	16.6 [14.9,18.4]	39.8 [37.5,42.1]	29.4 [27.3,31.6]	14.3 [12.7,16.0]
Total	5,086	100	17.7 [16.4,19.0]	39.0 [37.4,40.7]	29.4 [27.9,31.0]	13.8 [12.7,15.1]
**Nationality**						
Swiss	4,216	76.0	18.6 [17.2,20.0]	39.3 [37.5,41.1]	29.9 [28.2,31.6]	12.3 [11.2,13.5]
Non-Swiss	870	24.0	15.0 [12.3,18.1]	38.2 [34.3,42.3]	28.1 [24.5,32.0]	18.7 [15.7,22.0]
Total	5,086	100	17.7 [16.4,19.0]	39.0 [37.4,40.7]	29.4 [27.9,31.0]	13.8 [12.7,15.1]
**No. of household members**						
1	760	18.1	18.1 [14.9,21.9]	36.4 [32.2,40.9]	31.4 [27.4,35.8]	14.0 [11.3,17.2]
2	1,854	35.5	19.5 [17.4,21.7]	37.8 [35.1,40.6]	29.9 [27.4,32.5]	12.7 [11.0,14.7]
3-5	2,347	43.7	16.2 [14.4,18.1]	41.2 [38.7,43.7]	28.2 [26.0,30.5]	14.4 [12.7,16.3]
6 persons and more	125	2.8	15.9 [9.3,25.7]	36.5 [27.2,47.0]	29.5 [20.7,40.1]	18.1 [11.1,28.1]
Total	5,086	100	17.7 [16.4,19.0]	39.0 [37.4,40.7]	29.4 [27.9,31.0]	13.8 [12.7,15.1]
**Marital status**						
Single	1,669	35.3	13.0 [11.1,15.2]	44.8 [41.8,47.9]	30.0 [27.3,32.9]	12.2 [10.3,14.3]
Married	2,733	50.9	19.8 [18.0,21.7]	37.2 [35.0,39.5]	28.1 [26.1,30.2]	14.8 [13.3,16.6]
Widowed	150	3.1	22.7 [15.3,32.4]	22.2 [15.3,31.0]	34.0 [25.6,43.6]	21.0 [14.6,29.4]
Divorced	534	10.7	21.9 [17.9,26.6]	33.1 [28.4,38.1]	32.3 [27.8,37.2]	12.7 [9.7,16.4]
Total	5,086	100	17.7 [16.4,19.0]	39.0 [37.4,40.7]	29.4 [27.9,31.0]	13.8 [12.7,15.1]
**Biological children**						
Yes	2,968	57.6	19.6 [17.9,21.4]	36.9 [34.8,39.1]	28.8 [26.8,30.8]	14.7 [13.3,16.4]
No	2,074	42.4	15.1 [13.3,17.1]	41.8 [39.1,44.5]	30.5 [28.0,33.1]	12.6 [11.0,14.5]
Missing	44					
Total	5,086	100	17.7 [16.4,19.0]	39.0 [37.3,40.7]	29.5 [28.0,31.1]	13.8 [12.7,15.1]
**Language region**						
German	2,257	70.8	17.9 [16.3,19.6]	40.0 [37.9,42.2]	29.9 [28.0,32.0]	12.2 [10.8,13.7]
French	1,366	24.5	17.3 [15.3,19.5]	35.7 [33.0,38.4]	28.3 [25.7,30.9]	18.8 [16.6,21.1]
Italian	1,463	4.7	16.9 [15.0,19.1]	41.7 [39.0,44.3]	27.9 [25.6,30.4]	13.5 [11.7,15.5]
Total	5,086	100	17.7 [16.4,19.0]	39.0 [37.4,40.7]	29.4 [27.9,31.0]	13.8 [12.7,15.1]
**Urban/rural municipality**						
Urban	3,104	61.0	17.5 [15.9,19.3]	39.5 [37.3,41.7]	29.5 [27.5,31.6]	13.4 [12.0,15.0]

For peer review only

Intermediary	1,086	21.6	17.5 [15.0,20.3]	38.8 [35.3,42.4]	28.7 [25.6,32.0]	15.0 [12.6,17.8]
Rural	896	17.3	18.6 [15.7,21.9]	37.6 [33.8,41.6]	30.0 [26.4,33.8]	13.8 [11.3,16.8]
Total	5,086	100	17.7 [16.4,19.0]	39.0 [37.4,40.7]	29.4 [27.9,31.0]	13.8 [12.7,15.1]
**Education**						
Compulsory education or less	385	8.4	12.1 [8.7,16.6]	28.0 [22.8,33.8]	24.5 [19.6,30.2]	35.4 [29.7,41.5]
Upper secondary education	3,394	65.2	17.4 [15.9,19.0]	37.6 [35.5,39.6]	30.8 [28.9,32.8]	14.3 [12.9,15.8]
Tertiary education	1,283	26.4	20.1 [17.5,23.0]	46.2 [42.8,49.7]	27.8 [24.8,30.9]	5.9 [4.6,7.6]
Missing	24					
Total	5,086	100	17.7 [16.4,19.0]	39.1 [37.4,40.8]	29.5 [27.9,31.1]	13.8 [12.7,15.0]
**Religiousness**						
Very much	695	13.0	16.5 [13.3,20.2]	38.1 [33.6,42.8]	27.2 [23.3,31.6]	18.2 [14.9,22.0]
Somewhat	2,217	43.6	18.2 [16.3,20.2]	35.4 [33.0,38.0]	31.2 [28.8,33.6]	15.2 [13.4,17.2]
Not at all	2,138	43.5	17.7 [15.7,19.8]	42.8 [40.2,45.4]	28.5 [26.2,31.0]	11.0 [9.5,12.7]
Missing	36					
Total	5,086	100	17.7 [16.5,19.1]	39.0 [37.3,40.7]	29.5 [28.0,31.1]	13.8 [12.6,15.0]
**Working in health sector?**						
Yes	1,010	20.2	17.7 [15.0,20.8]	44.7 [40.9,48.6]	28.9 [25.5,32.5]	8.6 [6.7,11.0]
No	4,063	79.8	17.7 [16.3,19.2]	37.6 [35.8,39.5]	29.6 [27.9,31.4]	15.0 [13.7,16.4]
Missing	13					
Total	5,086	100	17.7 [16.5,19.1]	39.1 [37.4,40.8]	29.5 [27.9,31.1]	13.7 [12.6,14.9]
**Health status**						
Very unhealthy	61	0.8	20.9 [10.2,37.9]	28.1 [15.5,45.4]	26.0 [12.5,46.3]	25.1 [12.5,44.0]
Somewhat unhealthy	115	2.0	13.5 [7.9,22.2]	29.8 [20.6,41.1]	26.4 [17.5,37.7]	30.3 [21.2,41.2]
Neutral	661	13.5	17.5 [14.4,21.2]	33.0 [28.7,37.6]	31.0 [26.8,35.5]	18.5 [15.1,22.5]
Somewhat healthy	2,578	47.1	18.7 [16.8,20.7]	36.6 [34.3,39.0]	31.1 [28.9,33.4]	13.6 [12.1,15.4]
Very healthy	1,643	36.6	16.7 [14.6,18.9]	45.1 [42.2,48.0]	27.1 [24.6,29.8]	11.1 [9.4,13.0]
Missing	28					
Total	5,086	100	17.7 [16.4,19.0]	39.0 [37.4,40.7]	29.5 [27.9,31.1]	13.8 [12.6,15.0]
**Type of response**						
Web-based	3,519	71.0	17.6 [16.1,19.2]	41.7 [39.7,43.7]	29.0 [27.2,30.9]	11.7 [10.4,13.1]
Paper-based	1,567	29.0	17.9 [15.6,20.5]	32.0 [29.1,35.1]	30.6 [27.7,33.6]	19.5 [17.1,22.0]
Total	5,086	100	17.7 [16.4,19.0]	39.0 [37.4,40.7]	29.4 [27.9,31.0]	13.8 [12.7,15.1]

Figure 1 around here

Preferences for data storage

When asked how they preferred their data to be stored, most respondents preferred their data or samples to be anonymised (52.0%, 95% CI: 50.3% - 53.8%) or stored and used in coded form (43.4%, 95% CI: 41.7% - 45.1%). Only a few respondents (4.5%, 95% CI: 3.9% - 5.3%) preferred their data be stored and used in an identifiable form (Figure 2). Respondents who preferred coded or identifiable data and sample storage were more willing to hypothetically donate data or samples (aRP: 1.57, 95% CI: 1.47 – 1.68; aRP: 1.67, 95% CI: 1.47 – 1.89) than those who preferred anonymised samples. Many respondents who would like their data to be

For peer review only

stored anonymously indicated a preference to be re-contacted for each new project (36.8%, 95% CI: 34.5% - 39.2%) or re-contacted depending on the new project type (26.5%, 95% CI: 24.4% - 28.6%).

Figure 2 around here

Information participants need before deciding to donate data and samples to a biobank

We asked all respondents to endorse the top three pieces of information they would need to decide whether to donate data and samples to a biobank (Figure 3). While nearly all options had the endorsement of at least 25.0% of participants, the most relevant information were "the exact types of research which will be conducted" (57.1%) and "who has access to my data and samples" (48.3%). Only a few participants indicated that none of this information would help them decide (7.4%). Respondents were more willing to hypothetically provide data or samples when they endorsed any of the following information: "the exact types of research which will be conducted", "the potential benefits and risks of donating my data and samples", "the way data and samples are stored, such as made anonymous, encoded or stored together with my name", and "the security measures to keep data and samples private and protected". The answer options "who will benefit from the research" and "none of this information would help me decide" were negatively associated with willingness to donate data or samples to a biobank.

Figure 3 around here

Importance of financial or other material compensation for data donation

Most respondents indicated that money or other material compensation would not/would rather not be important (56.9%) for deciding whether to participate in a publicly funded Swiss biobank. Remaining participants were split, rating compensation as either moderately (24.9%) or rather/very important (18.3%). The greater importance a respondent placed on financial compensation, the less willing this person was to report hypothetical willingness to participate in a biobank (aRP: rather not important: 1.24, 95% CI: 1.14 – 1.34; very important: 0.79, 95% CI: 0.67 – 0.92).

Preferences for data ownership

Nearly half of respondents indicated that they should personally own the data and samples donated to a biobank (49.5%, 95% CI: 47.8% – 51.2%). Other potential 'data owners' included

For peer review only - http://bmjopen.bmj.com/site/about/guidelines.xhtml

universities involved with the biobank (11.0%, 95% CI: 10.0% - 12.1%), the biobank (10.7%, 95% CI: 9.7% - 11.8%), specific researchers who make discoveries (9.5%, 95% CI: 8.5% - 10.5%) or the Swiss government (5.4%, 95% CI: 4.7% - 6.3%). Participants who felt that ownership should belong to the biobank (aRP: 1.32, 95% CI: 1.21 – 1.45), the Swiss government (aRP: 1.23, 95% CI: 1.08 – 1.39), universities involved with the biobank (aRP: 1.36, 95% CI: 1.24 – 1.48) and specific researchers who make discoveries (aRP: 1.31, 95% CI: 1.18 – 1.45) were significantly more likely to be willing to hypothetically participate in a biobank, compared to persons who preferred that data ownership belong to them personally. Those that endorsed ownership by 'no one' (6.2%, 95% CI: 5.4% - 7.1%) were less likely to express willingness to participate (aRP: 0.76, 95% CI: 0.63 – 0.92).

Views on responsibility for data management and storage

Concerning data management and storage, most respondents preferred governance by the biobank (i.e. the management board, 34.3%, 95% CI: 32.7% - 35.9%) or an independent expert committee (e.g. independent researchers: scientists and clinicians not associated with the biobank, 30.7%, 95% CI: 29.1% - 32.3%). Those who favoured an independent committee representing the public (e.g. citizens, patients, the public) (8.0%) were less willing to participate in a hypothetical biobank (aRP: 0.78, 95% CI: 0.67 – 0.91) compared with respondents preferring governance by the biobank.

Trusted actors

Figure 4 shows the extent to which respondents trusted different actors to keep their data and samples confidential and protected if they had access to them in a biobank. Most respondents reported the strongest trust in their own doctor (76.3%), medical doctors in general (41.9%) and researchers at a university (41.7%). Health insurers (8.4%), pharmaceutical companies (6.4%), other for-profit companies from Switzerland (3.4%) and other global for-profit companies (2.0%) are trusted least.

Reported trust in researchers differed by level of education. Those with tertiary education reported stronger trust in researchers at a university (aRP: 1.26, 95% CI: 1.15 – 1.39) or researchers at other public institutes (aRP: 1.53, 95% CI: 1.34 – 1.75) than did those with an upper secondary education. Trust in health insurers differed by language region, education, religion, and health status: respondents living in the Italian-speaking region (aRP: 0.56, 95% CI: 0.42 – 0.75) of Switzerland trusted health insurance companies significantly less than respondents from the German-speaking region. Trust in health insurers decreased as

For peer review only - http://bmjopen.bmj.com/site/about/guidelines.xhtml

educational level increased (compulsory education or less: aRP: 1.51, 95% CI: 1.07 – 2.14; tertiary education: aRP: 0.67, 95% CI: 0.47 – 0.94), but increased as self-reported religiosity decreased (somewhat religious: aRP: 0.68, 95% CI: 0.51 – 0.92; not at all religious: aRP: 0.45, 95% CI: 0.32 – 0.63). Finally, healthier respondents' trust in health insurers was lower than for persons reporting a very unhealthy health status (somewhat healthy: aRP: 0.33, 95% CI: 0.14 – 0.75; very healthy: aRP: 0.40, 95% CI: 0.17 – 0.92).

Strong trust in eight of the nine mentioned institutions (see Figure 4) increased willingness to participate in a hypothetical personalised health research study or biobank. Only trust in global private, for-profit companies did not influence willingness to participate in such research. Respondents also distinguished between whether for-profit companies are from Switzerland or not: respondents with strong trust in Swiss for-profit companies were more willing to participate in personalised health research (aRP: 1.33, 95% CI: 1.18 – 1.51) compared with respondents with low trust in these companies. Those with compulsory education or less were more likely to trust Swiss for-profit companies (aRP: 2.29, 95% CI: 1.35 – 3.85) than those with secondary or tertiary education.

Figure 4 around here

DISCUSSION

This survey assessed the preferences of the Swiss public regarding management and governance of health data and biological samples in biobanks for personalised health research. We found that a majority of respondents hypothetically prefer anonymous or coded data storage in a biobank (52% and 43% respectively), yet also prefer to be re-contacted for each new project that will use their data (39%) or state that their preference depends on the type of project being considered (29%). Material compensation is not important in exchange for providing data and samples to a Swiss publicly-funded biobank, but nearly 50% prefer to own their own data. Respondents preferred that biobanks (34%) and independent expert committees (31%) be responsible for biobank governance. And finally, Swiss most trust their own doctor, doctors in general and university researchers to keep data confidential and protected, while for-profit companies (both global and Swiss), pharmaceutical companies and health insurances are not highly trusted.

These results highlight the Swiss public's desire to be in charge of how their data and samples are used, based on their preferences for study-specific consent and personal ownership of data or samples. Switzerland strongly emphasizes individual autonomy and citizen participation

For peer review only - http://bmjopen.bmj.com/site/about/guidelines.xhtml

[18], with direct democracy as a core feature of the Swiss political system. Therefore, these preferences are consistent with traditional cultural perceptions about individual control. However, as providing study-specific consent is often technically demanding and resource-intensive, most Swiss university hospitals have introduced broad consent ('general consent' in Switzerland) [19]. Patients are asked once for consent to use their anonymised or coded data and samples for unspecified future studies.

Across the globe, consent preferences for biobank research have been well studied, with divergent results. Whereas some studies of U.S. biobanks confirm that certain types of consent increase willingness to participate in research [20], others do not support this finding [21–23]. Most studies, however, identify a preference for broad consent [10], broad or study-specific consent [24], or finding both types of consent unacceptable [4,7]. In contrast, a pan-European study found that 67% prefer study-specific consent, with only 24% opting for broad consent [25]. Public preferences depend on context and culture. They are influenced by how questions are framed, which population is included (general population, research participants, patients), and the unique cultural norms and values of each country. Country-specific variation of preferences is therefore to be expected. Our finding that most Swiss respondents preferred study-specific informed consent is consistent with previously published European data [25]. It reflects the above-mentioned cultural preference to maintain a level of control when deciding for which studies data may be used [20]. It is noteworthy that existing literature about types of consent and data management is mostly U.S.-based, underscoring the importance of studying public preferences in other countries.

A further notable finding is that Swiss respondents who preferred their data be stored anonymously also frequently indicated a wish to be re-contacted for each new project (34.9%). With anonymised data, the connection to re-contact will be eliminated; therefore re-identifying participants is no longer possible. Such re-identification is, however, possible when data is stored in a coded form (sometimes called pseudoanonymized data). Conflicting preferences for anonymous data storage and a wish to control data use indicate a potential lack of sufficient public knowledge about data handling processes. Our survey asked about consent and storage preferences in two distinct, unlinked questions (first about consent, then about storage). Thus, we do not have insight into which choice the public would make between having their data stored anonymously and being able to re-consent to each study using their data or samples. Future research should assess preferences more specifically. However, a requirement for re-consent years after the original consent will likely result in selection bias. For example, some participants will have died and thus would be unable to re-consent.

For peer review only - http://bmjopen.bmj.com/site/about/guidelines.xhtml

Respondents in our study trust health professionals and institutions they know, namely their own doctors, universities and Swiss companies. They expressed the least trust in pharmaceutical companies, global for-profit companies and health insurance companies. Other studies assessing public opinions globally [26] and in the U.K., U.S., Australia, Canada and Singapore [2,27–32] confirm that individuals distinguish between research conducted by public and private actors. Our study identified a further distinction regarding for-profit companies: strong trust in Swiss for-profit companies increased willingness to participate in personalised health research, whereas trust in international for-profit companies had no influence. Recent scandals involving data sharing by international for-profit companies may help to account for this distrust; in 'Project Nightingale', for example, a privately-held U.S. hospital chain shared medical records with Google without notifying data subjects [33]. A similar debate occurred in summer 2021 when the British NHS planned to collect, pool and share patient data held by general practitioners, as part of the so-called General Practice Data for Planning and Research (GPDPR) [34]. The initiative was designed as an automatic opt-in process with a six-week opt-out window, and no option to delete data retrospectively. Data included medical records, information on sex, ethnicity, postal code, and week and year of birth (making data easily re-identifiable), and was to be shared with healthcare and research organisations, as well as commercial entities. After its announcement, the GPDPR was widely criticised as a "complete failure to develop a wide-ranging and far-reaching public engagement plan to communicate with the population" and was put on hold [34]. NHS Digital then initiated a consultation process and a public information campaign, to precede implementation of the initiative. This example highlights how engaging the public and ensuring the opportunity to make an informed choice about health data are crucial for sustainable and trusted health data use.

Given these insights, we might ask: how can Swiss biobanks better meet the needs and expectations of the public, and promote trust in this type of research? A first step is increasing transparency around governance mechanisms and information about research with health data and samples. We found that respondents who were unwilling to provide data or samples for research were also unsure of their preferred type of consent (80%) and were more likely to prefer their data be stored anonymously (58%). A lack of understanding of how data and samples are used for research might thus explain this disinterest in participation. It is essential that biobanks and other institutions collecting health data and samples provide accessible and understandable information via websites and brochures about how data can be reused, and the advantages and disadvantages of different types of consent for the individual and society. This would also help educate about the types of data storage, e.g. anonymous vs. coded data and

For peer review only

how data type influences the ability to be re-contacted or receive individual research results. Communication about future studies would promote participant knowledge about the use of their data and samples, and could help address perceived uncertainty associated with data donation and reuse, allowing participants a sense of meaningful control over their data and samples [35]. To alleviate this, Switzerland may wish to further consider developing dynamic consent models [16], which allow participants to personalise their consent preferences. Our data suggest that dynamic consent models better mirror the preferences of the Swiss public and permit participants to align future use of their data with their personal values. Such a move towards more granular, interactive consent models allows participants to retain greater control over their data and samples than is possible with broad consent alone [35].

Next, strengthening trust in biobanks and research initiatives will require increased transparency about how and with whom data is shared for research. Biobanks should specify for which projects data and samples will be used to any degree known, and maintain transparency about the range of future potential research that may occur, including whether data could be shared with the private sector, such as with pharmaceutical or medical technology companies [31]. In addition to listing all actors who have access to public data, the rationale and details of the cooperation should also be made transparent. In this way, potential data donors can decide for themselves whether they agree to such potential use of their data or samples before giving their consent.

As a way to increase trust, biobanks should treat the decision to donate data and samples as a process, rather than a one-time signature of the consent form [7]. Given that respondents in our study and other studies [27,30] frequently trust their own doctors most, biobanks and research initiatives should consider the possibility of doctors playing a primary role in informing patients about donating data or samples [36]. Shifting the responsibility and first point of contact to primary care would have the additional advantage of promoting broader awareness. Public trust is a dynamic construct [37,38]. It can be easily weakened if biobanks and research institutions do not meet the expectations that initially made them trustworthy in the public's perspective [39,40]. The systemic oversight approach can yield helpful mechanisms for building and maintaining public trust in increasingly complex research initiatives [35]. It requires that oversight mechanisms are adaptive to treat different data sources, and flexible to assess each intended use. Dynamic monitoring should be responsive to containing risks for data subjects.

As previously described [3], one limitation of our study is that this survey of Swiss residents only achieved a 34% response rate. However, with the full sampling list, we could construct non-response weights by all sociodemographic characteristics (age, sex, regions, and

For peer review only - http://bmjopen.bmj.com/site/about/guidelines.xhtml

languages). Such weighting reduces the potential non-response bias, making the results reasonably generalisable to the Swiss population. In addition, this survey obtained respondents' opinions at a single point in time. As we noted elsewhere [3], a longitudinal study could evaluate how opinions and views on this topic change over time. A further limitation of this survey is the general nature of the questionnaire, without specific examples of how the topics might apply in research; as such, respondents might not fully understand what is at stake in different scenarios when providing data or samples for research. Providing examples of different types of research in a future questionnaire could give respondents a better understanding of the dilemmas and trade-offs inherent in certain data donation contexts. For this report, we collapsed a 5-point Likert scale for levels of trust (1 = no trust to 5 = strong trust) to binary variables (low trust, strong trust). This approach will have removed nuances of respondents' attitudes towards different actors. And finally, as previously noted, we asked about the type of consent and storage preferences in two separate questions, preventing us from determining which was more important to our respondents.

CONCLUSIONS

This study reveals what the Swiss public expects from data management and governance when donating health data or biological samples for personalised health research. This study aimed to close the research gap on the Swiss public's preferences for data storage, consent, ownership and management, while also measuring trust in the ability of different healthcare actors to keep data and samples confidential and protected. These insights into public preference for data management and governance make it possible for Swiss biobanks and research institutions to consider the expectations and preferences of potential data donors. Aligning governance strategies with public expectations not only promotes trust in biobank endeavours [41]; the resulting trust also positively influences public willingness to participate in health research and biobanks [42]. Transparent communication about research, data use, and implementation of consent models where participants can choose how often they would like to be asked for permission to use their data (dynamic consent) are key to increasing the willingness of individuals in Switzerland to provide their data to biobanks.

For peer review only

Ethics approval: The Ethics Committee of ETH Zurich (EK 2018-N-66) approved the study. By responding to the survey, participants provided their informed consent.

Acknowledgments: We thank members at the Health Ethics & Policy Lab at ETH Zurich for discussions on the questionnaire development and pre-testing, Christoph Freymond from the Federal Office for Statistics for providing the data sample, Stefan Wehrli and his team from the Decision Sciences Laboratory at ETH Zurich for setting up and maintaining the Call Center and data collection infrastructure, Jan Kaiser for automating the digitalisation of the paper-based questionnaires and Shannon Hubbs for proofreading this manuscript. We especially thank the respondents who took part in the survey.

Competing interests: None declared.

Funding: This work was supported by Swiss National Science Foundation (SNSF) Grant 157556 to E.V. and through the personal research fund of E.V. at ETH Zurich, which covered the costs of data collection. M.E. was supported by special project funding (Grant 189498) from the SNSF.

Author Contributions: Conceptualization, C.Be., M.Z., M.E., E.V.; methodology, C.Br., C.Be., M.Z., E.V., M.E., K.E.O.; software, C.Be.; validation, C.Br., C.Be., M.Z., K.E.O.; formal analysis, C.Br., C.Be., M.Z.; investigation, C.Br., C.Be., M.Z., E.V., M.E., K.E.O.; resources, E.V.; data curation, C.Br., C.Be.; writing—original draft preparation, C.Br.; writing—review and editing, C.Br., C.Be., M.Z., E.V., M.E., K.E.O.; visualization, C.Br., C.Be.; supervision, M.Z., E.V., K.E.O.; project administration, C.Br.; funding acquisition, M.E., E.V. All authors have read and agreed to the published version of the manuscript.

Data Sharing Statement: Dataset available from ETH Zurich, DOI: 10.3929/ethz-b-000474690.

For peer review only

REFERENCES

1 Flores M, Glusman G, Brogaard K, *et al.* P4 medicine: How systems medicine will transform the healthcare sector and society. *Per Med* 2013;**10**:565–576. doi:10.2217/pme.13.57

2 Vidgen ME, Kaladharan S, Malacova E, *et al.* Sharing genomic data from clinical testing with researchers: public survey of expectations of clinical genomic data management in Queensland, Australia. *BMC Med Ethics* 2020;**21**:1–11. doi:10.1186/s12910-020-00563-6

3 Brall C, Berlin C, Zwahlen M, *et al.* Public willingness to participate in personalized health research and biobanking: A large-scale Swiss survey. *PLoS One* 2021;**16**:e0249141. doi:10.1371/journal.pone.0249141

4 Tomlinson T, De Vries R, Ryan K, *et al.* Moral concerns and the willingness to donate to a research biobank. *JAMA - J Am Med Assoc* 2015;**313**:417–9. doi:10.1001/jama.2014.16363

5 Clayton EW, Halverson CM, Sathe NA, *et al.* A systematic literature review of individuals' perspectives on privacy and genetic information in the United States. *PLoS One* 2018;**13**:1–26. doi:10.1371/journal.pone.0204417

6 Goddard KAB, Smith KS, Chen C, *et al.* Biobank Recruitment: Motivations for Nonparticipation. *Biopreserv Biobank* 2009;**7**:119–21. doi:10.1089/bio.2009.0006

7 De Vries RG, Tomlinson T, Kim HM, *et al.* Understanding the public's reservations about broad consent and study-by-study consent for donations to a biobank: Results of a national survey. *PLoS One* 2016;**11**:1–11. doi:10.1371/journal.pone.0159113

8 D'Abramo F, Schildmann J, Vollmann J. Research participants' perceptions and views on consent for biobank research: a review of empirical data and ethical analysis. *BMC Med Ethics* 2015;**16**:1–11. doi:10.1186/s12910-015-0053-5

9 Smith ME, Sanderson SC, Brothers KB, *et al.* Conducting a large, multi-site survey about patients' views on broad consent: challenges and solutions. *BMC Med Res Methodol* 2016;**16**:1–11. doi:10.1186/s12874-016-0263-7

10 Garrison NA, Sathe NA, Antommaria AHM, *et al.* A systematic literature review of individuals' perspectives on broad consent and data sharing in the United States. *Genet Med* 2016;**18**:663–71. doi:10.1038/gim.2015.138

11 Grady C, Eckstein L, Berkman B, *et al.* Broad Consent for Research With Biological Samples: Workshop Conclusions. *Am J Bioeth* 2015;**15**:34–42. doi:10.1080/15265161.2015.1062162

For peer review only - http://bmjopen.bmj.com/site/about/guidelines.xhtml

12 Dankar FK, Gergely M, Dankar SK. Informed Consent in Biomedical Research. *Comput Struct Biotechnol J* 2019;**17**:463–74. doi:10.1016/j.csbj.2019.03.010

13 Wendler D. Broad versus Blanket Consent for Research with Human Biological Samples. *Hastings Cent Rep* 2013;**43**:3–4. doi:10.1002/hast.200

14 Blasimme A, Brall C, Vayena E. Reporting Genetic Findings to Individual Research Participants: Guidelines From the Swiss Personalized Health Network. *Front Genet* 2020;**11**. doi:10.3389/fgene.2020.585820

15 Scheibner J, Ienca M, Kechagia S, *et al.* Data protection and ethics requirements for multisite research with health data: A comparative examination of legislative governance frameworks and the role of data protection technologies. *J Law Biosci* 2020;**7**:1–30. doi:10.1093/jlb/lsaa010

16 Meier-Abt P, Lawrence AK, Selter L, *et al.* The Swiss approach to precision medicine. *Swiss Med Wkly* 2018;:1–8.

17 Zou G. A Modified Poisson Regression Approach to Prospective Studies with Binary Data. *Am J Epidemiol* 2004;**159**:702–6. doi:10.1093/aje/kwh090

18 Biller-Andorno N, Zeltner T. Individual Responsibility and Community Solidarity — The Swiss Health Care System. *N Engl J Med* 2015;**373**:2193–7. doi:10.1056/NEJMp1508256

19 Universitäre Medizin Schweiz. Generalkonsent für die Forschung. https://www.unimedsuisse.ch/de/projekte/generalkonsent (accessed 9 May 2021).

20 Goodman D, Bowen D, Wenzel L, *et al.* The Research participant perspective related to the conduct of genomic cohort studies: A systematic review of the quantitative literature. *Transl Behav Med* 2018;**8**:119–29. doi:10.1093/tbm/ibx056

21 Kaufman DJ, Baker R, Milner LC, *et al.* A survey of U.S adults' opinions about conduct of a nationwide Precision Medicine Initiative® cohort study of genes and environment. *PLoS One* 2016;**11**:1–14. doi:10.1371/journal.pone.0160461

22 Sanderson SC, Brothers KB, Mercaldo ND, *et al.* Public Attitudes toward Consent and Data Sharing in Biobank Research: A Large Multi-site Experimental Survey in the US. *Am J Hum Genet* 2017;**100**:414–27. doi:10.1016/j.ajhg.2017.01.021

23 Gayet-Ageron A, Rudaz S, Perneger T. Biobank attributes associated with higher patient participation: A randomized study. *Eur J Hum Genet* 2016;**25**:31–6. doi:10.1038/ejhg.2016.132

24 Ewing AT, Erby LAH, Bollinger J, *et al.* Demographic differences in willingness to provide broad and narrow consent for biobank research. *Biopreserv Biobank*

For peer review only - http://bmjopen.bmj.com/site/about/guidelines.xhtml

2015;**13**:98–106. doi:10.1089/bio.2014.0032

25 Gaskell G, Gottweis H, Starkbaum J, *et al.* Publics and biobanks: Pan-European diversity and the challenge of responsible innovation. *Eur J Hum Genet* 2013;**21**:14–20. doi:10.1038/ejhg.2012.104

26 Middleton A, Milne R, Almarri MA, *et al.* Global Public Perceptions of Genomic Data Sharing: What Shapes the Willingness to Donate DNA and Health Data? *Am J Hum Genet* 2020;**107**:743–52. doi:10.1016/j.ajhg.2020.08.023

27 Ghafur S, Van Dael J, Leis M, *et al.* Public perceptions on data sharing: key insights from the UK and the USA. *Lancet Digit Heal* 2020;**2**:e444–6. doi:10.1016/S2589-7500(20)30161-8

28 IPSOS MORI for Wellcome Trust. The One-Way Mirror: Public attitudes to commercial access to health data Report prepared for the Wellcome Trust. 2016.

29 Trinidad SB, Fullerton SM, Bares JM, *et al.* Genomic research and wide data sharing: Views of prospective participants. *Genet Med* 2010;**12**:486–95. doi:10.1097/GIM.0b013e3181e38f9e

30 Milne R, Morley KI, Howard H, *et al.* Trust in genomic data sharing among members of the general public in the UK, USA, Canada and Australia. *Hum Genet* 2019;**138**:1237–46. doi:10.1007/s00439-019-02062-0

31 Braunack-Mayer A, Fabrianesi B, Street J, *et al.* Sharing Government Health Data With the Private Sector: Community Attitudes Survey. *J Med Internet Res* 2021;**23**:e24200. doi:10.2196/24200

32 Lysaght T, Ballantyne A, Toh HJ, *et al.* Trust and Trade-Offs in Sharing Data for Precision Medicine: A National Survey of Singapore. *J Pers Med* 2021;**11**:921. doi:10.3390/jpm11090921

33 Copeland R. Google's 'Project Nightingale' Gathers Personal Health Data on Millions of Americans. Wall Str. J. 2019.https://www.wsj.com/articles/google-s-secret-project-nightingale-gathers-personal-health-data-on-millions-of-americans-11573496790 (accessed 7 Jun 2021).

34 Burki T. Concerns over England's new system for collecting general practitioner data. *Lancet Digit Heal* 2021;**3**:e469–70. doi:10.1016/S2589-7500(21)00148-5

35 Vayena E, Blasimme A. Health research with big data: Time for systemic oversight. *J Law, Med Ethics* 2018;**46**:119–29. doi:10.1177/1073110518766026

36 Kelly SE, Spector TD, Cherkas LF, *et al.* Evaluating the consent preferences of UK research volunteers for genetic and clinical studies. *PLoS One* 2015;**10**:1–12.

For peer review only

doi:10.1371/journal.pone.0118027

37 Gille F, Brall C. Can we know if donor trust expires? About trust relationships and time in the context of open consent for future data use. *J Med Ethics* 2021;:medethics-2020-106244. doi:10.1136/medethics-2020-106244

38 Kerasidou A. The role of trust in global health research collaborations. *Bioethics* 2019;**33**:495–501. doi:10.1111/bioe.12536

39 Shabani M, Bezuidenhout L, Borry P. Attitudes of research participants and the general public towards genomic data sharing: A systematic literature review. *Expert Rev Mol Diagn* 2014;**14**:1053–65. doi:10.1586/14737159.2014.961917

40 Gille F, Axler R, Blasimme A. Transparency about Governance Contributes to Biobanks' Trustworthiness: Call for Action. *Biopreserv Biobank* 2021;**19**:83–5. doi:10.1089/bio.2020.0057

41 Dive L, Critchley C, Otlowski M, *et al.* Public trust and global biobank networks. *BMC Med Ethics* 2020;**21**:1–9. doi:10.1186/s12910-020-00515-0

42 Middleton A, Milne R, Almarri MA, *et al.* Global Public Perceptions of Genomic Data Sharing: What Shapes the Willingness to Donate DNA and Health Data? *Am J Hum Genet* 2020;**107**:1–10. doi:10.1016/j.ajhg.2020.08.023

For peer review only

For peer review only - http://bmjopen.bmj.com/site/about/guidelines.xhtml

Legends

Figure 1. Overview of how often the biobank should ask for permission to re-use the data (type of consent). (**a**) Overall percentages of respondents who preferred various options regarding how often the biobank should ask for permission to re-use the data (type of consent). “Ask me only once” corresponds to a ‘broad consent’ approach, and “Ask me again for every new project” corresponds to specific consent and/or a dynamic consent approach. (**b**) Percent of each response choice who are willing / not willing to participate in personalised health research.

Figure 2. Preferences for data and sample storage. (**a**) Percentages of respondents who preferred each approach to data and sample storage; (**b**) Percent of each response choice who are willing / not willing to participate in personalised health research.

Figure 3. Preferences for information necessary in order to make a decision to donate data and samples to a biobank. Figure presents population weighted percentages; respondents were asked to indicate their top three informational preferences. * indicates statistical difference between the two groups of respondents willing and not willing to participate, $p<0.05$.

Figure 4. Trust in actors to keep data confidential and protected. Figure presents population weighted percentages of total respondents.

For peer review only

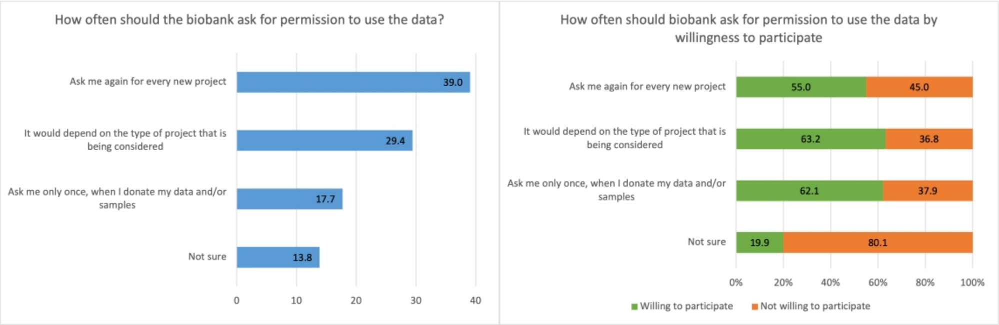

Figure 1. Overview of how often the biobank should ask for permission to re-use the data (type of consent). (a) Overall percentages of respondents who preferred various options regarding how often the biobank should ask for permission to re-use the data (type of consent). "Ask me only once" corresponds to a 'broad consent' approach, and "Ask me again for every new project" corresponds to specific consent and/or a dynamic consent approach. (b) Percent of each response choice who are willing / not willing to participate in personalized health research.

290x94mm (330 x 330 DPI)

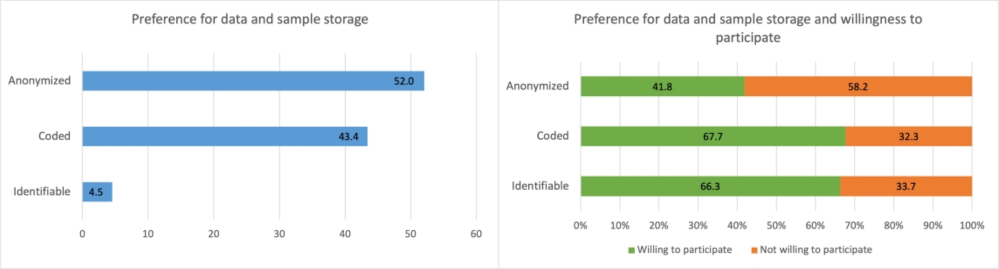

Figure 2. Preferences on data and sample storage. (a) Percentages of respondents who preferred each approach to data and sample storage; (b) Percent of each response choice who are willing / not willing to participate in personalized health research.

284x76mm (330 x 330 DPI)

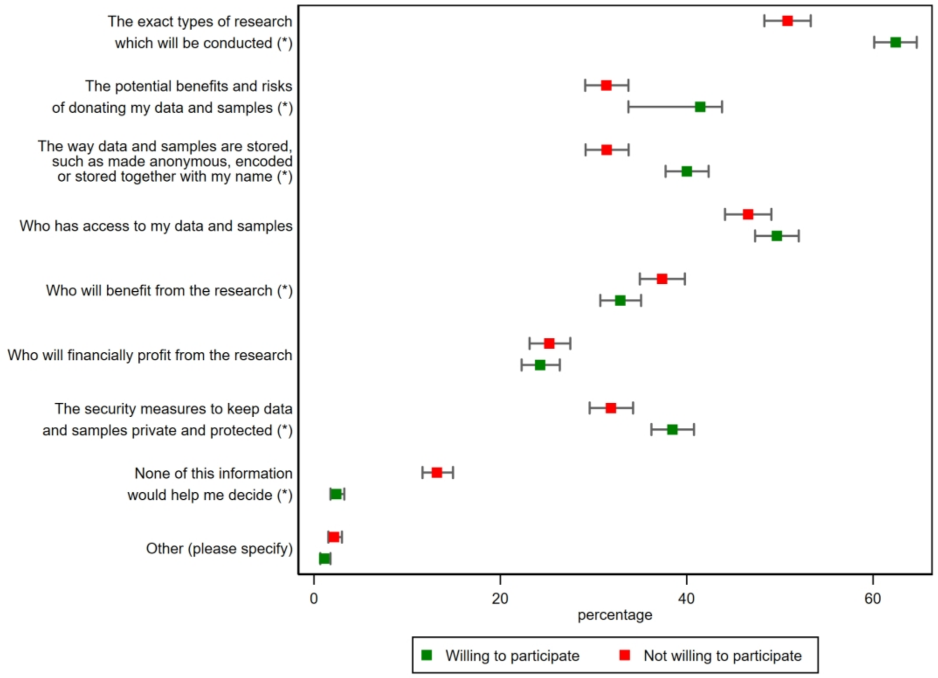

Figure 3. Preferences for information necessary in order to make a decision to donate data and samples to a biobank. Figure presents population weighted percentages; respondents were asked to indicate their top three informational preferences. * indicates statistical difference between the two groups of respondents willing and not willing to participate, p<0.05.

705x513mm (72 x 72 DPI)

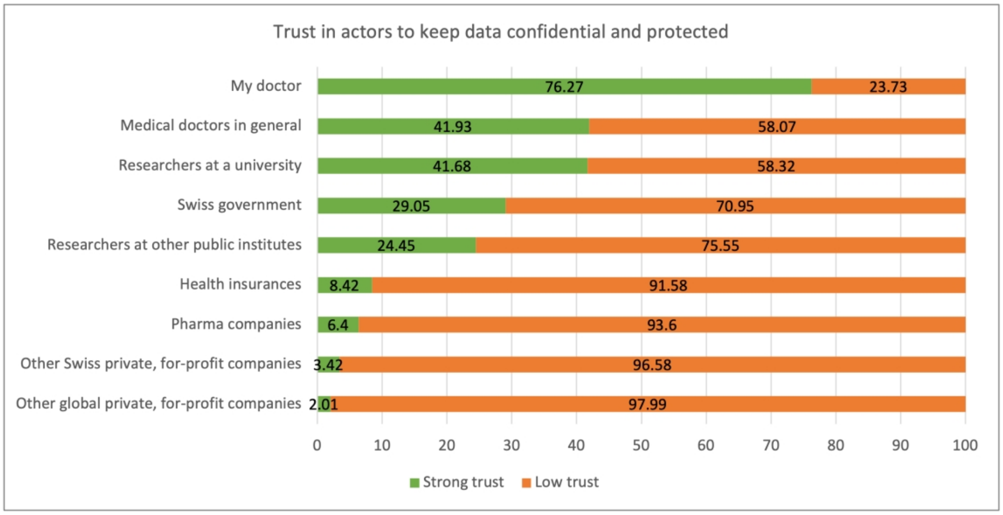

Figure 4. Trust in actors to keep data protected. Figure presents population weighted percentages of total respondents.

189x96mm (330 x 330 DPI)

STROBE Statement—Checklist of items that should be included in reports of ***cross-sectional studies***

	**Item No**	**Recommendation**	**Page No**
**Title and abstract**	1	(*a*) Indicate the study's design with a commonly used term in the title or the abstract	1
		(*b*) Provide in the abstract an informative and balanced summary of what was done and what was found	2
**Introduction**			
Background/rationale	2	Explain the scientific background and rationale for the investigation being reported	3-4
Objectives	3	State specific objectives, including any prespecified hypotheses	4
**Methods**			
Study design	4	Present key elements of study design early in the paper	4
Setting	5	Describe the setting, locations, and relevant dates, including periods of recruitment, exposure, follow-up, and data collection	4-5
Participants	6	(*a*) Give the eligibility criteria, and the sources and methods of selection of participants	4
Variables	7	Clearly define all outcomes, exposures, predictors, potential confounders, and effect modifiers. Give diagnostic criteria, if applicable	5
Data sources/ measurement	8*	For each variable of interest, give sources of data and details of methods of assessment (measurement). Describe comparability of assessment methods if there is more than one group	5
Bias	9	Describe any efforts to address potential sources of bias	5
Study size	10	Explain how the study size was arrived at	4
Quantitative variables	11	Explain how quantitative variables were handled in the analyses. If applicable, describe which groupings were chosen and why	5
Statistical methods	12	(*a*) Describe all statistical methods, including those used to control for confounding	5
		(*b*) Describe any methods used to examine subgroups and interactions	5
		(*c*) Explain how missing data were addressed	5
		(*d*) If applicable, describe analytical methods taking account of sampling strategy	5
		(*e*) Describe any sensitivity analyses	5
**Results**			
Participants	13*	(a) Report numbers of individuals at each stage of study—eg numbers potentially eligible, examined for eligibility, confirmed eligible, included in the study, completing follow-up, and analysed	4, reported elsewhere (Brall et al., 2021)
		(b) Give reasons for non-participation at each stage	4, reported elsewhere (Brall et al., 2021)

For peer review only - http://bmjopen.bmj.com/site/about/guidelines.xhtml

		(c) Consider use of a flow diagram	/
Descriptive data	14*	(a) Give characteristics of study participants (eg demographic, clinical, social) and information on exposures and potential confounders	6-10
		(b) Indicate number of participants with missing data for each variable of interest	4, reported elsewhere (Brall et al., 2021)
Outcome data	15*	Report numbers of outcome events or summary measures	6-13
Main results	16	(*a*) Give unadjusted estimates and, if applicable, confounder-adjusted estimates and their precision (eg, 95% confidence interval). Make clear which confounders were adjusted for and why they were included	6-13
		(*b*) Report category boundaries when continuous variables were categorized	6-13
		(*c*) If relevant, consider translating estimates of relative risk into absolute risk for a meaningful time period	/
Other analyses	17	Report other analyses done—eg analyses of subgroups and interactions, and sensitivity analyses	6-13
**Discussion**			
Key results	18	Summarise key results with reference to study objectives	13-14
Limitations	19	Discuss limitations of the study, taking into account sources of potential bias or imprecision. Discuss both direction and magnitude of any potential bias	17
Interpretation	20	Give a cautious overall interpretation of results considering objectives, limitations, multiplicity of analyses, results from similar studies, and other relevant evidence	14-16
Generalisability	21	Discuss the generalisability (external validity) of the study results	17
**Other information**			
Funding	22	Give the source of funding and the role of the funders for the present study and, if applicable, for the original study on which the present article is based	18

*Give information separately for exposed and unexposed groups.

Note: An Explanation and Elaboration article discusses each checklist item and gives methodological background and published examples of transparent reporting. The STROBE checklist is best used in conjunction with this article (freely available on the Web sites of PLoS Medicine at http://www.plosmedicine.org/, Annals of Internal Medicine at http://www.annals.org/, and Epidemiology at http://www.epidem.com/). Information on the STROBE Initiative is available at www.strobe-statement.org.

For peer review only
